# *Rindera graeca* (A. DC.) Boiss. & Heldr. (Boraginaceae) In Vitro Cultures Targeting Lithospermic Acid B and Rosmarinic Acid Production

**DOI:** 10.3390/molecules28124880

**Published:** 2023-06-20

**Authors:** Katarzyna Sykłowska-Baranek, Małgorzata Gaweł, Łukasz Kuźma, Beata Wileńska, Mateusz Kawka, Małgorzata Jeziorek, Konstantia Graikou, Ioanna Chinou, Ewa Szyszko, Piotr Stępień, Patryk Zakrzewski, Agnieszka Pietrosiuk

**Affiliations:** 1 Department of Pharmaceutical Biology, Faculty of Pharmacy, Medical University of Warsaw, 1 Banacha St., 02-097 Warsaw, Poland; mgawel1@wum.edu.pl (M.G.); mateusz.kawka@wum.edu.pl (M.K.); mjeziorek@wum.edu.pl (M.J.); pryzmik16@op.pl (E.S.); piotrste1@wp.pl (P.S.); patrol20013@gmail.com (P.Z.); agnieszka.pietrosiuk@wum.edu.pl (A.P.); 2 Department of Biology and Pharmaceutical Botany, Faculty of Pharmacy, Medical University of Łódź, 1 Muszyńskiego, 90-151 Łódź, Poland; lukasz.kuzma@umed.lodz.pl; 3 Faculty of Chemistry, University of Warsaw, 1 Pasteura St., 02-093 Warsaw, Poland; bwilenska@chem.uw.edu.pl; 4 Biological and Chemical Research Centre, 101 Żwirki i Wigury St., 02-097 Warsaw, Poland; 5 Laboratory of Pharmacognosy and Chemistry of Natural Products, Faculty of Pharmacy, National and Kapodistrian University of Athens, Panepistimiopolis, 15771 Athens, Greece; kgraikou@pharm.uoa.gr (K.G.); ichinou@pharm.uoa.gr (I.C.)

**Keywords:** antioxidants, phenolic compounds, micropropagation, hairy roots, anatomical roots, bioreactor, RAPD, SCoT

## Abstract

The in vitro cultures of *Rindera graeca*, a rare endemic plant, were developed as a sustainable source of phenolic acids. Various shoot and root cultures were established and scaled up in a sprinkle bioreactor. A multiplication rate of 7.2 shoots per explant was achieved. HPLC–PDA–ESI–HRMS analysis revealed the presence of rosmarinic acid (RA) and lithospermic acid B (LAB) as the main secondary metabolites in both the shoot and root cultures. The maximum RA (30.0 ± 3.2 mg/g DW) and LAB (49.3 ± 15.5 mg/g DW) yields were determined in root-regenerated shoots. The strongest free radical scavenging activity (87.4 ± 1.1%), according to 2,2-diphenyl-1-picrylhydrazyl-hydrate assay, was noted for roots cultivated in a DCR medium. The highest reducing power (2.3 µM ± 0.4 TE/g DW), determined by the ferric-reducing antioxidant power assay, was noted for shoots cultivated on an SH medium containing 0.5 mg/L 6-benzylaminopurine. A genetic analysis performed using random amplified polymorphic DNA and start codon targeted markers revealed genetic variation of 62.8% to 96.5% among the investigated shoots and roots. This variability reflects the capacity of cultivated shoots and roots to produce phenolic compounds.

## 1. Introduction

The demand for plant-derived compounds for pharmaceutical and cosmetic industries is constantly increasing, accompanied by the growing threat of over-exploitation of natural resources. One solution to this great challenge may be found in plant biotechnology methods that allow high yields of bioactive compounds to be obtained in an environmentally independent manner while protecting ecosystem biodiversity [1,2,3].

Among the many and varied plant secondary metabolites, phenolic compounds are known to exert numerous beneficial effects on human health, including preventing cancer, diabetes, chronic degenerative diseases, and cardiovascular diseases, all of which are recognized to be associated with oxidative stress [4,5]. This efficacy of phenolic compounds is considered to be connected with their antioxidant potential, as they can delay, inhibit, or prevent biomolecule oxidation by, e.g., scavenging free radicals [6].

**Figure 1:**
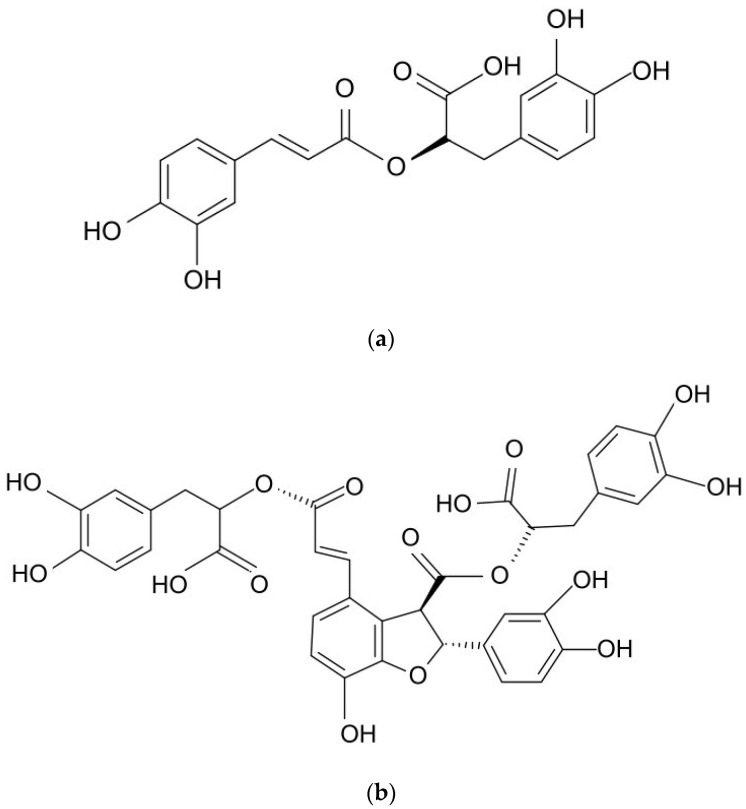
Chemical structures of (**a**) rosmarinic acid (RA) and (**b**) lithospermic acid B (LAB).

Previous studies have detailed a wide range of biotechnological approaches that can enhance the production of phenolic compounds via the cultivation of various plant parts, cells, tissues, and organs [7,8,9]. Although the biotechnological production of rosmarinic acid (RA; Figure 1a) has been intensively investigated, relatively few sources have examined the biotechnological efforts leading to enhanced production of lithospermic acid B (LAB; Figure 1b), also known as salvianolic acid B. Widely distributed in the plant kingdom, RA was found in all plants of the Boraginaceae family [10]. It is an ester of caffeic acid (CA) and 3,4-dihydroxyphenyllactic acid, while LA is a conjugate of RA and CA, and LAB is a dimer of RA [11,12].

*Rindera graeca* (A. DC.) Boiss. & Heldr. (synonyms: *Cynoglossum graecum* (A. DC.) Greuter & Burdet and *Mattia graeca* A. DC., Ref. [13]) belongs to the tribe Cynoglossae of Boraginaceae [14,15]. The genus *Rindera* comprises about 25 species that are predominantly distributed in Central and Western Asia [16]. *R. graeca* is a Greek endemic mountain species that grows from Peloponnesus (southern Greece) and Sterea Ellada toward North Pindos (northwest Greece) [17] on rocky slopes at altitudes of 1500–2300 m. In 1997, *R. graeca* was included in the IUCN Red List of Threatened Plants and marked as a rare species [18]. A few reports have examined the traditional usage of some *Rindera* species in Iranian and Turkish folk medicine, e.g., the seeds of *R. lanata* (Lam.) Bunge used to treat joint pain [19] and *R*. *lanata* and *R. caespitosa* (DC.) Gürke roots used as a poultice due to their anti-inflammatory properties [20]. Furthermore, a methanolic extract prepared from the aerial parts (leaves, stem, and flowers) of *R. lanata* was found to demonstrate antiviral activity against three different human rotavirus strains [21]. The authors noted the presence of twelve phenolic compounds, of which the major constituents were malic, quinic, and rosmarinic acids. In addition, essential oils distilled from aerial parts of *R. lanata* var. canescens demonstrated moderate antimicrobial potential [22]. In the chemical profile of the aerial parts of naturally grown *R. graeca*, nine phenolic compounds, including CA and its derivatives (chlorogenic acid, rabdosiin (a tetramer of CA), and its disodium salt), RA, salvianolic A acid, and LAB, were determined [23]. Later, these naturally occurring secondary metabolites were also identified in in vitro-cultivated *R. graeca* shoots and roots [24]. These were accompanied by rinderol, a furano-naphthoquinone, which was isolated for the first time from *Onosma paniculata* root extract by Dong et al. [25] and later from *Cynoglossum columnae* root in vitro cultures [26]. Next, the extract profiling of extracts derived from in vitro-cultivated *R. graeca* roots of different origins subjected to cold or drought stress was performed by our group using the HPLC–PDA–ESI–HRMS method, along with the quantitative determination of CA, RA, and LAB acids [27]. This study revealed that the major metabolite in all extracts was LAB.

The aim of the present study was to establish a system for the micropropagation of the rare, endemic boraginaceous species *R. graeca* to obtain plant material efficiently producing biologically active phenolic compounds, especially rosmarinic acid and lithospermic acid B. The genetic diversity of the cultivated shoot and root lines was assessed using RAPD (random amplified polymorphic DNA) and SCoT (start codon targeted) markers, and the antioxidant properties of the extracts obtained via biotechnological methods were evaluated. It also proposes a scale-up approach in the sprinkle bioreactor. This is the first detailed report on the establishment of *R. graeca* in vitro cultures for the production of phenolic compounds.

## 2. Results and Discussion

### 2.1. Micropropagation

Of the 30 *R. graeca* seeds used to initiate the in vitro culture, only three germinated. Two plantlets were used to micropropagate *R. graeca*, and the other served as the starting material for the initiation of callus tissue. The callus obtained on an MS medium supplemented with 0.5 mg/L NOA was transferred to a liquid hormone-free MS medium, and adventitious root development was observed within four weeks. To initiate the shoot cultures, the roots derived from the seed plantlets were cut off, and the shoots were placed on a DCR medium supplemented with 0.5 mg/L BAP. Next, micropropagation was carried out on various DCR medium modifications (Table S1) to induce auxiliary shoots. There are no data on the seed germination of this rare endemic species. However, for *R. umbellate*, it has been reported that its propagation in nature is limited by fungal infection and insufficient seed maturity [28]. The phenomenon of strong seed dormancy is not rare among Boraginaceous species. The strong dormancy at maturity of the seeds, probably maintained for a long time, has been described for *Heliotropium europaeum*, for example [29]. Another reason for limited seed germination may be the low viability of the seeds and the hard seed coat, as was reported for *Lithospermum canescens* [30].

**Figure 2:**
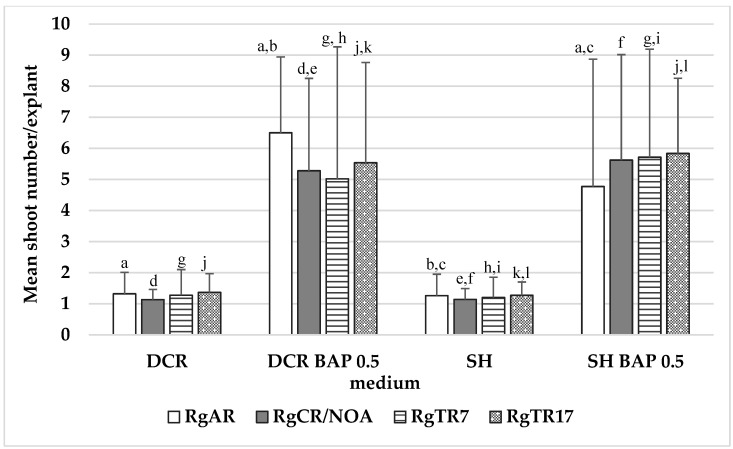
The number of auxiliary shoots formed per explant in *Rindera graeca* shoot cultures. Shoots were obtained via regeneration from roots and were cultivated on DCR and SH without growth regulators or supplementation with 0.5 mg/L BAP. Values represent means ± SD, and those marked with the same letter are statistically significant (*p* < 0.05). RgAR—shoots regenerated from anatomical roots; RgCR/NOA—shoots regenerated from auxin-induced roots; RgTR7—shoots regenerated from RgTR7 hairy roots; RgTR17—shoots regenerated from RgTR17 hairy roots.

The highest shoot induction rates were noted on the DCR medium supplemented with 0.5 mg/L BAP (DCR/BAP) and DCR/SV with the addition of (DCR/SV/KIN): 7.2 ± 3 and 7.1 ± 5, respectively (Figure 2).

**Figure 3:**
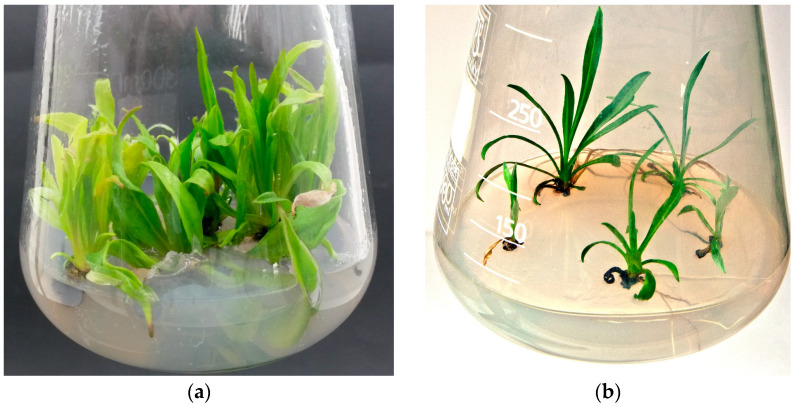
(**a**) Rosette of *Rindera graeca* shoots (RgS line) forming on DCR medium supplemented with 0.5 mg/L BAP; (**b**) roots spontaneously developing at the bases of RgCR/NOA line shoots cultivated on hormone-free DCR medium.

However, the quality of shoots developed on the DCR/BAP medium was superior to that formed on DCR/SV/KIN, in which the leaves in the rosettes were light green to yellow-green, which could be attributed to the reduced amount of salts and vitamins in the DCR/SV/KIN medium compared with the full-strength one (DCR). On the DCR/BAP medium, the shoots formed luxuriant rosettes composed of deep green leaves (Figure 3a). The approach of multiple shoot induction via direct organogenesis applied in the current study resulted in the growth of seven new shoots within four weeks, which seemed to be satisfactory [31]. When placed on the hormone-free DCR medium, the shoots spontaneously developed roots at their bases (Figure 3b).

During the cultivation of both non-transformed and hairy roots, spontaneous shoot regeneration was observed; these were used to initiate cultures of regenerated shoots on solid DCR or SH media, which were plain or supplemented with 0.5 mg/L BAP. The presence of cytokinin was essential for shoot regeneration from the roots. The highest multiplication rate was noted for shoots regenerated from RgAR roots and cultivated on the DCR/BAP medium, which amounted to 6.5 ± 2.4 shoots per explant. The regenerants demonstrated a significantly lower (*p* < 0.05) multiplication rate than the shoots derived from seedlings (Figure 2). In addition, when placed on the hormone-free DCR medium, the shoots spontaneously developed roots at their bases (Figure 3b).

No studies so far have examined the micropropagation of *R. graeca* from existing meristems or its regeneration from roots; however, one paper examined the in vitro multiplication of *R. umbellata* (Waldst. & Kit.) Gürke [31]. The authors used immature embryos as the starting material for this; 72% germinated successfully and were efficiently propagated on an MS medium containing 1.0 mg/L BAP and 0.5 mg/L indole-3-acetic acid (IAA) supplemented with 0.1 M sucrose, followed by acclimatization to the greenhouse and field conditions. However, the authors found that the type of sugar and its concentration were the major factors influencing the number of explants forming buds, the most optimal values being 0.06 M sucrose or 0.3 M glucose.

In the reported up-to-date micropropagation protocols developed for Boraginaceous plants via direct organogenesis, the highest multiplication rates were associated with the use of thidiazuron (TDZ). However, TDZ does not appear to be the only treatment with beneficial effects on shoot regeneration and proliferation in Boraginaceous plants. In Table 1, the efforts that have been undertaken to obtain the highest micropropagation rates are summarized.

### 2.2. Root Cultures

**Figure 4:**
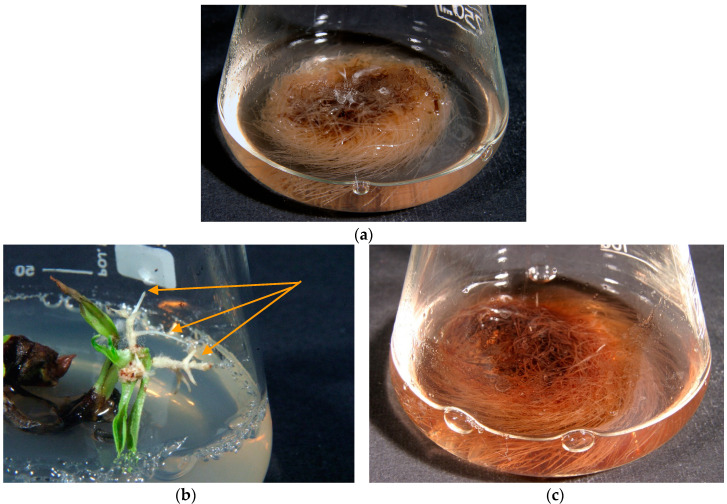
(**a**) *Rindera graeca* anatomical roots growing in liquid hormone-free SH media; (**b**) hairy roots emerging from *R. graeca* leaves (indicated by arrows) inoculated with the *Agrobacterium rhizogenes* ATCC 15834 strain after 4 weeks of culture; (**c**) hairy roots of RgTR17 line cultivated in liquid hormone-free DCR medium for 4 weeks.

The *R. graeca* roots developed spontaneously on the RgS line shoots following transfer onto the hormone-free DCR medium. The roots were cut off and used to establish anatomical root RgAR cultures (Figure 4a). The second auxin-induced RgCR/NOA root line was initiated from roots developed on callus tissue induced by the addition of NOA 0.5 mg/L to the MS medium.

**Figure 5:**
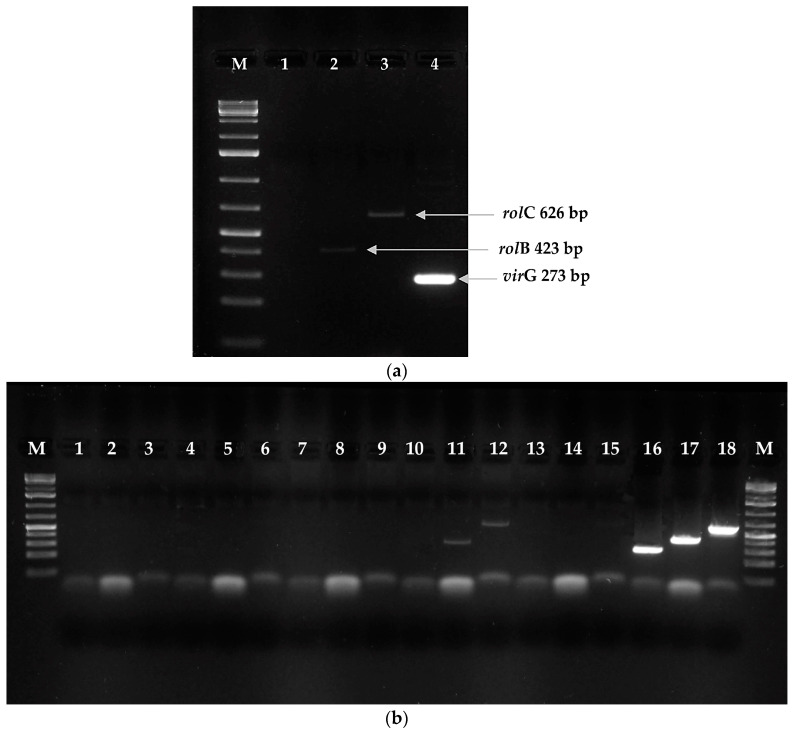
Confirmation of genetic transformation by PCR analysis of genomic DNA isolated from *Rindera graeca* shoots regenerated from roots. (**a**) RgTR17 line: M—molecular weight marker (GeneRulerTM 1 kb Plus DNA ladder); lane 1—*vir*G; lane 2—*rol*B (423 bp); lane 3—*rol*C (626 bp); lane 4—*Agrobacterium rhizogenes* 15834 plasmid—*vir*G (273 bp). (**b**) M—molecular weight marker GeneRulerTM 100 bp DNA ladder; lanes 1–6: RgAR shoot line; lanes 7–9: RgCR/NOA shoot line; lanes 10–12: RgTR7 shoot line: 10—*vir*G, 11—*rol*B (423 bp), 12—*rol*C (626 bp); lanes 13–15: RgCR/NOA shoot line; lanes 16–18: *A. rhizogenes* 15834 plasmid: lane 16—*vir*G (273 bp), lane 17—*rol*B (423 bp), and lane 18—*rol*C (626 bp).

Following of transformation the using *Agrobacterium rhizogenes* ATCC 15834 strain, 60 roots emerged within two to four weeks after explant infection (Figure 4b). Finally, five hairy root line cultures were established on the basis of visual assessment. During the three subsequent passages, two root lines were chosen for further investigations on the basis of root growth capacity: RgTR7 and RgTR17 (Figure 4c). PCR analysis confirmed the incorporation of the *rol*B and *rol*C genes into RgTR7 and RgTR17 roots (Figure 5).

**Figure 6:**
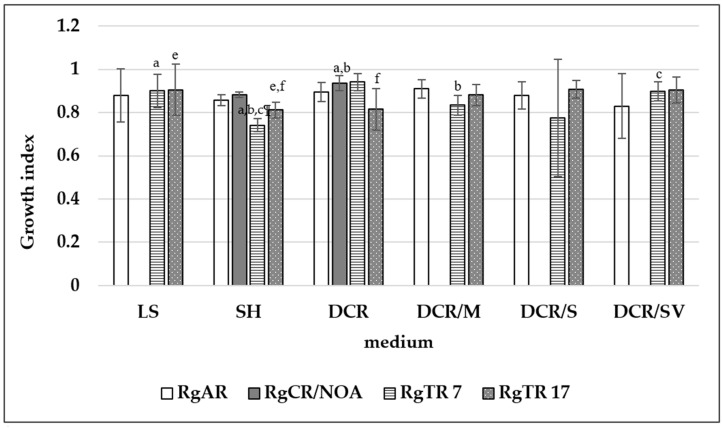
Growth index of *Rindera graeca* non-transformed and hairy roots. Means denoted with the same letter are statistically significant (*p* < 0.05).

**Figure 7:**
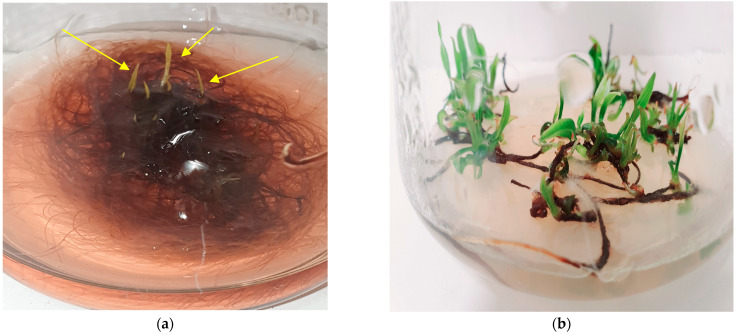
Spontaneously regenerating shoots (indicated by arrows) form hairy roots of RgTR17 line during cultivation in the dark on liquid hormone-free DCR medium, 4th week of culture (**a**); shoots regenerating form explants of RgTR17 hairy root line when grown on solid SH supplemented with 0.5 mg/L BAP and cultivated under a 16/8h (light/dark) photoperiod, 4th week of culture (**b**).

The non-transformed root and hairy root lines were cultivated in LS, SH, and the four modifications of the DCR medium (Figure 6). The highest root growth index was noted for the auxin-induced RgCR/NOA and RgTR7 root lines cultivated in DCR medium: 0.94 ± 0.035 and 0.94 ± 0.039, respectively. The DCR medium was chosen for further investigation. During cultivation roots, irrespectively the root line, in liquid hormone-free DCR or SH media in the dark, spontaneous regeneration of the shoots was observed (Figure 7a). On the basis of this observation, the cultures of shoots regenerating from roots on solid media were established at 16 h/8 h (light/dark) photoperiod (Figure 7b).

### 2.3. Sprinkle Bioreactor Cultures

**Figure 8:**
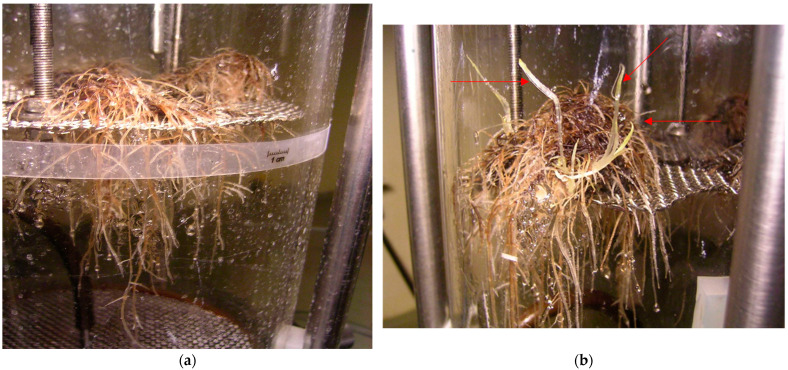
(**a**) RgTR17 hairy roots cultivated in mist bioreactor in SH hormone-free medium for 3 weeks; (**b**) spontaneously regenerated shoots (indicated by arrows).

The *R. graeca* root cultures were scaled up in a nutrient sprinkle bioreactor. In the first batch, the root line RgTR17 was cultivated in the DCR medium; the fresh biomass rose from 15.23 g to 26.41 g, and the dry biomass increased from 1.68 g to 4.23 g. These values represented 1.7- and 2.5-fold increases in growth, respectively; this biomass growth was achieved as a result of 40 s/80 s intervals of medium sprinkle/pause. By contrast, the fresh biomass of roots increased from 2.35 g to 37.50 g (16-fold), and the dry biomass increased from 0.29 g to 4.83 g (16.7 times) with a 15 s/30 s interval of medium sprinkle/pause. The RgTR7 hairy root line was also cultivated in the DCR medium under 15 s/30 s sprinkle/pause intervals; however, these roots did not grow. As a result, the medium was changed from DCR to SH. Under these new conditions, i.e., the SH medium, the RgTR17 line roots demonstrated higher growth capacities than the RgTR7 roots (Figure 8a); their fresh and dry biomass increased 8.5- and 9.5-fold, while the RgTR7 roots increased 6.3- and 8.3-fold, respectively. The RgTR17 roots demonstrated 1.4-fold enhanced growth in the SH medium compared with the DCR medium. The RgAR roots were cultivated in the SH medium and demonstrated 9.8- and 7.6-fold increases in fresh and dry biomass, respectively. In addition, spontaneous shoot regeneration was observed during culture in the SH medium, irrespective of the root line (Figure 8b). The fresh and dry biomass of the regenerants were 17.6 g and 2.2 g for shoots derived from the roots of the RgTR17 line, 21.4 g and 2.5 g for the RgTR7 line, and 76.28 g and 10.65 g for the RgAR line. No shoot regeneration was observed when cultivating roots in the bioreactor using the DCR medium.

### 2.4. Genetic Analysis Using RAPD and SCoT Markers

**Figure 9:**
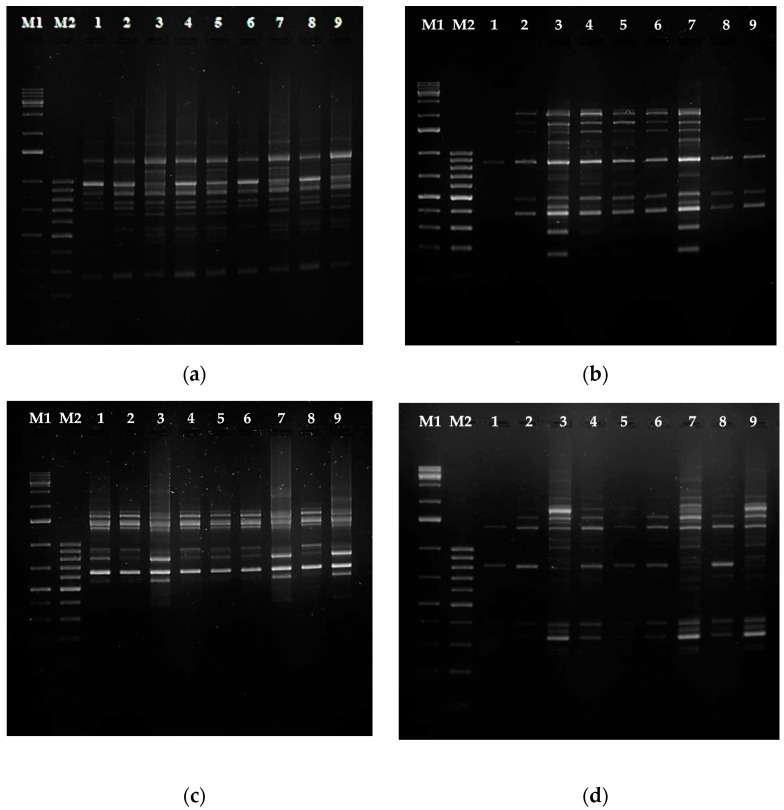
PCR analysis of genomic DNA isolated from *Rindera graeca* shoot and root lines: (**a**) RAPD primer OPA7; (**b**) RAPD primer OPA11; (**c**) SCoT17 primer; (**d**) SCoT30 primer; M1—molecular weight marker GeneRuler^TM^ 1 kb DNA ladder; M2—molecular weight marker GeneRuler^TM^ 100 bp DNA ladder; lane 1—RgS shoots, lane 2—RgAR shoots, lane 3—RgAR roots, lane 4—RgCR/NOA shoots, lane 5—RgCR/NOA roots, lane 6—RgTR7 shoots, lane 7—RgTR7 roots, lane 8—RgTR17 shoots, lane 9—RgTR17 roots.

Most studies on molecular markers applied to the identification of cultivars and varieties to date have focused on RAPD RFLP, SSRS, ISSR, or ALFP markers [40,41,42]. To assess genetic diversity, usually, two types of DNA primers are used, and we chose RAPD and SCoT ones. We expected that both types of primers would allow the generation of a sufficient number of bands to assess the genetic diversity among the shoot and root lines investigated in our study. In the RAPD analysis, 10 of the 20 primers (Table S2) showed well-resolved bands (Figure 9a,b). When SCoT primers were used to amplify all the genotypes, 11 of the 36 tested exhibited well-resolved bands (Figure 9c,d). Genetic analysis performed with RAPD and SCoT markers identified between 62.8% and 96.5% similarity depending on the regenerant shoot or root line (Table S3).

**Figure 10:**
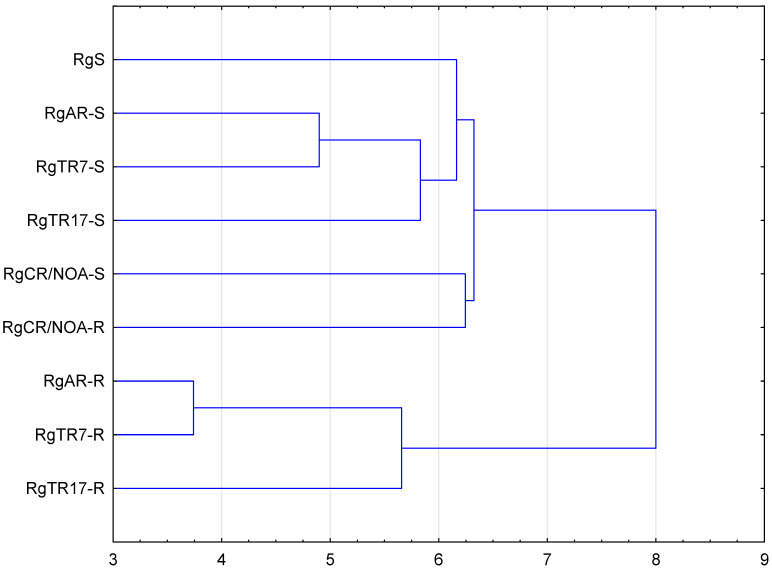
UPGMA agglomeration analysis of *Rindera graeca* shoots and roots cultivated in vitro: RgS—shoots derived from seeds; RgCR/NOA-S—shoots derived from RgCR/NOA roots; RgCR/NOA-R—auxin-induced roots regenerated from callus; RgTR7-S—transgenic shoots derived from RgTR7 hairy roots; RgTR7-R—hairy roots of the RgTR7 line; RgTR17-S—transgenic shoots derived from RgTR17 hairy roots; RgTR17-R—hairy roots of the RgTR17 line.

UPGMA analysis led to the identification of two major clusters. The first was divided into several sub-clusters; one of them comprised RgS, RgAR, RgCR/NOA, RgTR7, and RgTR17 shoots, while the other comprised RgCR/NOA shoots and roots. The second comprised roots of lines RgAR, RgTR7, and RgTR17 (Figure 10). Surprisingly, the hairy RgTR7 and anatomical RgAR root lines were found to be present in a common sub-cluster. The variation observed could have been caused by a combination of the genetic transformation and somaclonal variation imposed by the tissue culture conditions [43,44,45]. The RgCR/NOA root line was derived from calli obtained from a medium supplemented with NOA, and hence it is possible that the anatomical roots emerging from the base of the RgS shoots also appeared through some callus cells. It has been proposed that somaclonal variation could be connected to the presence of the callus stadium and changes in its ploidy levels; these have been reported to increase gradually during callus initiation but then return to low ploidy during prolonged cultivation [38,45]. Furthermore, it was described that in cultures of highly organized organs, such as stems, leaves, and roots, somaclonal variation is generated more often than in explants with existing meristems, i.e., shoot/root tips or lateral buds [46,47]. In hairy root lines, it is possible that the *ROL* genes of T-DNA, which could be randomly integrated with the plant genome, may be responsible for genetic variability [48]. This phenomenon could be also a result of chromosomal aberrations or gene silencing [49]. Many plant species have demonstrated spontaneous or induced shoot regeneration from hairy roots in vitro, as well as somatic embryogenesis [50]. An excised shoot can develop into a whole plant, which was also observed in the current study. A number of studies have reported regenerants from hairy roots developing hairy root syndrome, with the extent of the symptoms varying according to the number of incorporated *ROL* genes.

In the current study, the morphology of shoots regenerated from *R. graeca* hairy roots did not differ from that of the non-transformed shoots, and this was in accordance with the results of the genetic analysis (Figure 7b and Figure 9). No morphological traits characteristic of shoots developed from hairy roots were observed (Figure S1), e.g., wrinkled leaves or dwarf and bushy phenotypes with short leaves attributed to the incorporation of *ROL* genes from *A. rhizogenes* T-DNA [51,52]. The absence of phenotypic changes between hairy roots and their regenerants could be explained by the fact that the TL- and TR-DNA regions of T-DNA are presented in the agropine-type bacterial strain and could be independently transferred to the nuclei of an infected plant cell and differently integrated into the plant genome. The TL-DNA *ROL* genes are crucial for the induction of hairy root syndrome [51,52], and the occurrence of full hairy root symptoms requires the expression of *rol*A, *rol*B*,* and *rol*C genes (see [51] and references therein). In the current study, *rol*B and *rol*C genes were confirmed in both the hairy roots and the derived regenerants. Both genes are believed to be necessary to induce significant improvements in root growth, with *rol*B playing a key role in root formation and *rol*C stimulating lateral branching [51]. Although the presence of *rol*A was not checked, the morphological features of the roots and shoot indicated its absence, as no bushy plantlets with wrinkled leaves were observed. Similarly, the successful incorporation of *rol*B and *rol*C genes into the regenerants was demonstrated by the increased formation of adventitious roots; various changes in leaf morphology for *rol*B; and reduced flower size, altered leaf morphology (small with wrinkled edges), and enhanced formation of auxiliary shoots among others for *rol*C. None of the mentioned symptoms were observed in the regenerants in the present study, as all three *rol* genes are needed for the full hairy root phenotype to develop [51,52]. In addition, the lack of any visible morphological traits of the hairy root phenotype could be attributed to the weak expression of *rol*B and *rol*C in the current experiment (Figure 5), probably due to the position effect and promotor activity modulation, as reported by Schmülling et al. [51] and Mauro et al. [52]. Similarly, the hairy root lines RgTR7 and RgTR17 did not differ morphologically from the non-transformed lines, demonstrating similar low *ROL* gene expression to the regenerants.

### 2.5. Phenolic Compounds Production

Although several publications have examined the in vitro production of RA and/or LAB as well as various other phenolic compounds, in Boraginaceous plant cultures, the majority have employed cultures of undifferentiated cells, i.e., callus and suspension cultures [53,54,55,56,57,58,59,60,61]. The number of studies that have described the accumulation of RA and/or LAB in root cultures of Boraginaceae plants is limited. There is one report on *Eritrichum sericeum* (Lehm.) DC. adventitious root cultures [62] and another on *Lithospermum erythrorhizon* callus suspension hairy root cultures [63]. Most of the literature concerning RA and LAB production in plant cultures has been dedicated to various members of the Lamiaceae family [7,8,9]. However, our group recently reported a comprehensive analysis of changes in the phytochemical profiles of the *R. graeca* RgAR, RgTR7, and RgTR17 root lines subjected to drought and cold stress [27].

**Figure 11:**
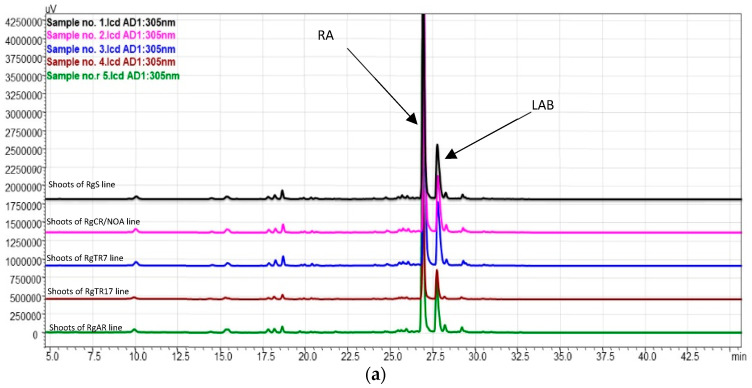

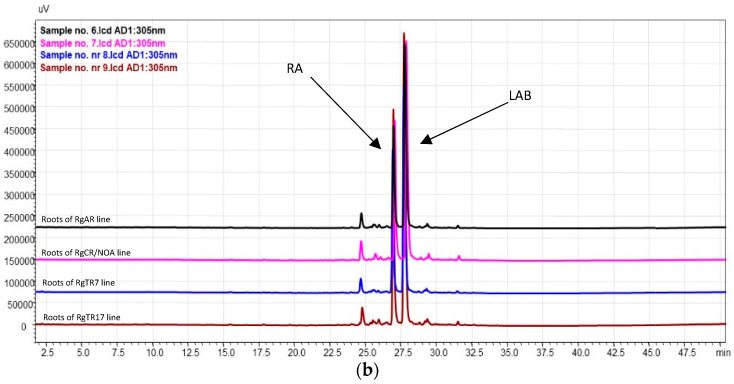
HPLC–PDA chromatograms (wavelength 305 nm) of (**a**) shoot samples and (**b**) root samples. RA—rosmarinic acid; LAB—lithospermic acid B.

In the present study, the presence of RA and LAB were confirmed in *R. graeca* shoots and roots cultivated in vitro in flasks and a sprinkle bioreactor by comparing their MS1 and MS2 fragmentation ion and retention times with the reference standards in negative ionization mode (Table 2, Figure 11, Table S4). LA was not detected.

These findings are consistent with those of Naliwajski et al. [27] and Graikou et al. [24], who investigated in vitro cultures of *R. graeca*, as well as those of Ganos et al. [23], who examined the aerial parts of wild-grown *R. graeca*.

The highest yield of RA was detected in shoots regenerated from RgAR roots during cultivation in the SH medium in the sprinkle bioreactor: 30.0 ± 3.2 mg/g DW. This value was over 1.5-fold higher than that in flask cultures of the same shoot line in the same medium and almost 2.4-fold more than that in RgS shoots (Table 2). Among all the investigated root lines, the maximum RA content was determined in the RgAR roots cultivated in flasks in the DCR medium (14.4 ± 5.9 mg/g DW). In the bioreactor, the highest yield of RA was detected in RgAR roots during cultivation in the SH medium (5.6 ± 0.7 mg/g DW) (Table 2). In addition, in our previous investigation on *R. graeca* root cultures, we observed differences in the RA biosynthetic capacities among three tested root lines: RgAR, RgTR7, and RgTR17. The highest RA content was determined in the hairy roots of the RgTR7 line (33.69 mg/g DW) [27].

Weremczuk-Jeżyna et al. [64] reported RA production of up to 18.7 mg/g DW and LAB production of up to 5.9 mg/g DW in *Dracocephalum forestii* (Lamiaceae) shoots cultivated in flask cultures and RA and LAB production of 17.9 and 6.5 mg/g DW, respectively, in a sprinkle bioreactor [65]. However, transformed shoots of *D. forestrii* accumulated higher amounts of RA of up to 24.80 mg/g DW [66].

RA can be detected in all parts of the intact plant. It can also be produced by biotechnological methods, with levels as high as 36% DW reported in suspension cultures of *Salvia officinalis* L. and up to 14% DW in *Ocimum basilicum* L. hairy root cultures (see [10] and references therein).

Thus, the results of the present study are consistent with those of previous reports.

LAB was detected in both the shoots and the roots (Table 2). Its highest yield (49.3 ± 15.5 mg/g DW) was determined in RgAR regenerants cultivated in the DCR/BAP medium. This yield was significantly higher than that of the other regenerants, including the RgS shoot line. In the cultures carried out in the bioreactor, LAB was detected only in regenerated shoots of RgAR and RgTR17 lines, and only when SH medium was applied; in such cases, the yield in RgAR was over threefold higher than in the RgTR17 regenerates.

The *R. graeca* hairy roots in the present study demonstrated a fourfold higher LAB yield (11.7 mg/g DW) and twofold higher RA yield (19.97 mg/g DW) compared with previously studied *D. forestii* hairy cultures [67]. However, the highest yield of LAB determined under the conditions of the current study was over nine times lower (11.7 versus 106.07 mg/g DW) than that reported previously by our group [27]. This may be attributed to differences in environmental conditions, including the in vitro culture maintenance protocol, as this is known to have a strong influence on the profile of secondary metabolites.

Similar results have been observed in in *Salvia miltiorrhiza* Bunge hairy root cultures, which demonstrated an increase in LAB content accompanied by a decrease in RA content (Table 2) [68], as well in *L. erythrorhizon* cell and hairy root cultures [56]. Fujita et al. [69] report an appreciably higher amount of LAB than RA in cultivated cells, with the choice of medium having a marked effect; in this case, LAB production was almost eightfold higher in production medium M9 compared with LS. In hairy root cultures, the authors observed a higher accumulation of LAB than RA, which was only present in trace amounts, as well as LA production. While RA was distributed mainly in the aerial parts, LAB and LA were observed in the underground ones. LA was not detected in the shoots. In the present study, the content of the investigated phenolic compounds also depended on the culture conditions. Higher RA concentrations were observed in the shoots during cultivation in the SH medium; however, higher RA concentrations were only observed in the roots when they were cultivated in the DCR medium (Table 2). LA was detected in both the shoot and the root cultures, although it was observed in the roots only when they were grown in the DCR medium. It is not surprising that both investigated phenolic compounds were observed in hairy roots, as they possess all the enzymes that mimic the biosynthetic pathways of the parent plant [70]. Modern biotechnology methods allow considerably higher RA and caffeic acid derivatives to be obtained from plant material cultivated in vitro than from parent plants. In hairy root cultures of *Dracocephalum kotschyi*, depending on the root line, the RA content ranged from 10 to 1500 µg/g DW, which was 15 times higher than that from the roots of the intact plant [71]. In *E. sericeum*, RA accumulation amounted to 2.04% DW in callus cultures and 4.50% DW in adventitious roots. In comparison, RA was only present at 0.07% DW in the aerial and underground parts of the native plant and at 1.10% DW in *L. erythrorhizon* callus, compared with 0.28% and 0.66% in the aerial and underground parts of the native plant [62]. Similarly, Bryukhanov et al. [59] reported a 112-fold higher content of RA in cell culture than in native *E. sericeum* plant roots. In addition, in *Martensia maritima*, another borage species, the level of RA amounted to 0.74% DW in the callus tissue, compared with 0.11%, 0.20%, and 0.16% DW in the stems, leaves, and roots of the native plant [59].

### 2.6. Antioxidant Capacity of Plant Extracts

Significantly higher total phenolic compound (TPC) content was determined in extracts derived from shoots cultivated in untreated SH or SH supplemented with BAP (Table 3) compared with other plant extracts. The highest TPC yield (up to 264.7 ± 32.8 GAE mg/g DW) was noted in RgCR/NOA shoots maintained in the SH/BAP medium. In the root cultures, significantly higher TPC was noted in roots cultivated in the SH medium than in DCR, with the highest concentration amounting to 45.7 ± 8.1 GAE mg/g DW in the RgAR line. The detected TPC levels were remarkably higher in the shoots than in the roots (Table 3). The antioxidant level of the *R. graeca* plant material was determined using DPPH and FRAP assays. In the former, DPPH radical scavenging by an antioxidant resulted in a loss of absorbance directly proportional to the potential of the antioxidant, while in the latter, a Fe^3+^ complex with tripyridyltriazine was reduced to an intense blue Fe^2+^ complex by an antioxidant in an acidic medium, with the depth of color indicating the strength of the antioxidant [72].

In the present study, the strongest DPPH free radical scavenging activity was noted for extracts prepared from roots cultivated in the DCR medium; among shoot cultures, the highest ability was observed for the extracts derived from shoots grown in the SH/BAP medium (Table 3). The antioxidant potential ranged from 32.8% to 87.4% depending on the root line and from 27.9% to 85.6% depending on the shoot line. The strongest reducing power determined in the FRAP assay was demonstrated for extracts prepared from shoots, which were also cultivated in the SH/BAP medium (Table 3). The estimated correlation between the DPPH radical scavenging activity and the TPC, RA, and LAB concentration amounted to r^2^ = 0.86, 0.42, and 0.41, respectively. By contrast, the results of the FRAP method demonstrated only a weak correlation with TPC (r^2^ = 0.16); however, higher correlations were observed with the RA and LAB content: r^2^ = 0.18 and −0.14, respectively. These findings indicate that the antioxidant potential determined by the DPPH and FRAP assays could be influenced by the presence of other groups of secondary metabolites reported for *R. graeca*, including flavonoids and shikonin-type naphthoquinones [24,27]. Although shikonin itself was not detected, four shikonin derivatives were previously reported [73]. Shikonin/alkannin and their derivatives, bearing both the quinone and hydroquinone moieties, are known to possess substantial antioxidant properties [74,75]. A structure–activity relationship study of selected shikonin/alkannin derivatives revealed that the side chain-OH group at C-1′ positively influenced the antioxidant capacity [76] and that a naphthoquinone moiety was essential for such activity [74]. Nevertheless, among the shoot cultures in the present study, significantly higher antioxidant activity was observed in extracts derived from cultures grown with BAP; these conditions offered a favorable multiplication rate (Figure 2) and shoot quality (Figure 3a). These findings are consistent with those reported in investigations of *R. graeca* aerial parts [23]. They are also similar to those found for in vitro *Mentha haplocalyx* cultures [23], in which both crude extracts and those derived from the shoots were subjected to phytochemical analysis. She et al. [23] isolated eight polyphenolic acids from the chlorophyll fraction of this species, among them LAB, RA, and LA, and demonstrated that LAB exerted higher DPPH scavenging activity than RA and LA.

This also indicates that a group of secondary metabolites other than investigated phenolic acids could a play role in the antioxidant power of the investigated extracts. This complex phenomenon was also described by Krolicka et al. [77] in in vitro *Drosera aliciae* Raym.-Hamet cultures.

## 3. Materials and Methods

### 3.1. Seed Germination

The seeds of *R. graeca* were collected and donated by Prof. Ioanna Chinou from the University of Athens in 2006, and the in vitro cultures were initiated the same year. Prior to sterilization, the seeds were placed at −20 °C for 72 h, and after sterilization, they were gently cut with a sterile scalpel. The seeds were surface-sterilized with 90% ethanol for 30 s and treated with 5% natrium hypochlorite for 30 min. Finally, they were rinsed three times with sterile water. A hormone-free, half-strength basal MS [78] medium (½ MS) containing 3% sucrose was used for the germination process. The pH of the medium was adjusted to 5.8, and it was autoclaved. Finally, 30 seeds of *R. graeca* were used to initiate the culture. This culture was carried out in 100 mL Erlenmeyer flasks containing 30 mL of the ½ MS solid medium at 25 ± 1 °C in the dark.

### 3.2. Shoot and Root Cultures

#### 3.2.1. Shoot Micropropagation

The explants used as the source for the experiments were four-week-old seedlings of *R. graeca*. Micropropagation was performed using solid DCR [79] with four modifications: full strength (DCR), half the level of macroelements (DCR/M), half-strength of macro- and microelements (DCR/S), and half-strength of basal salts and vitamins (DCR/SV). The media were supplemented with 6-benzylaminopurine (BAP) or kinetin at doses of 0.5 or 1 mg/L before sterilization. Three shoots per flask were placed into a 300 mL Erlenmeyer flask containing 50 mL of one of the modified DCR media described above. Each DCR modification was supplemented with 3% sucrose, and the pH was adjusted to 5.7 prior to autoclaving. The experiments were carried out over three subsequent four-week passages, and each group was composed of five flasks with four to five explants per flask. The shoot cultures were performed for four weeks with a 16 h/8 h (light/dark) photoperiod with light provided by cool-white fluorescent lamps (40 µM/m^2^/s) at a temperature of 25 ± 1 °C. The multiplication rate was calculated after the four-week culture and expressed as the number of newly developed auxiliary shoots per explant ± SD.

#### 3.2.2. Hairy Root Cultures

The four-week-old shoots were infected with *Agrobacterium rhizogenes* strain ATCC 15834, as described by Pietrosiuk et al. [80]. On the basis of the visual evaluation, out of 60 roots emerging from the inoculation sites, two hairy root lines were chosen for further investigation. After excision, to remove the bacteria from their tissues, the roots were placed into 100 mL Erlenmeyer flasks containing 30 mL of liquid hormone-free LS medium [81] supplemented with 3% sucrose and Cefotaxime (500 mg/L), and they were cultivated for two weeks. The pH of the medium was adjusted to 5.6 prior to autoclaving. This procedure was repeated three times. The putative hairy roots were cultivated in a liquid hormone-free LS medium to examine the growth parameters. For this purpose, the LS medium and the four DCR medium modifications were used. The roots were cultivated on a 105 rpm gyratory shaker (INFORS, Bottmingen, Switzerland) in 250 mL Erlenmeyer flasks containing 50 mL of the medium, with a subculture to fresh medium every four weeks; the incubation was performed at 23 ± 1 °C in the dark. To determine the growth of roots, they were gently pressed onto filter paper to remove the medium and weighted, and their fresh weight (FW) was recorded. The root growth index was calculated by subtracting the fresh weight at the time of inoculation (time zero) from that at the time of harvest (day 28th of culture) and dividing by the weight at the time of harvest. The growth index was calculated on the basis of the results of five subsequent passages (five flasks per medium variant). The material was later lyophilized under vacuum at 0.340 mbar and an initial product temperature of −20 °C (lyophilizer Christ ALPHA1-4 LSC, Osterode am Harz, Germany) until a constant weight was achieved, that is, 24–48 h, and weighed to determine the dry weight (DW). On the basis of the root biomass growth results, the DCR medium variant was chosen for further cultivation. However, after the sprinkle bioreactor experiments, the root growth was also investigated in an SH [82] liquid hormone-free medium (50 mL in 250 mL Erlenmeyer flasks) for three passages with five replicates. The SH medium was supplemented with 3% sucrose, and its pH was adjusted to 5.7 prior to autoclaving.

#### 3.2.3. Non-Transformed Root Cultures

Two non-transformed root cultures were established. The first one was initiated from roots that spontaneously developed at the bases of shoots cultivated on a solid hormone-free DCR medium over four weeks of culture (RgAR). The second root line was induced by auxin and originated from callus tissue obtained from one-week-old hypocotyls of *R. graeca* (RgCR/NOA). The callus culture was initiated by an MS medium supplemented with 0.5 mg/L 2-naphthoxyacetic acid (NOA) and 3% sucrose; the pH was adjusted to 5.8 prior to autoclaving. The callus tissue developed on the hypocotyl over four weeks of culture, and next was transferred to a liquid hormone-free MS medium. Root development was observed for four weeks of culture. The roots were then transferred to 250 mL Erlenmeyer flasks containing 50 mL of the liquid hormone-free DCR or SH medium (each supplemented with 3% sucrose, pH adjusted to 5.7 prior to autoclaving) for further cultivation. The cultures were maintained at 23 ± 1 °C in the dark at 105 rpm on an INFORS gyratory shaker. The growth index of auxin-induced roots was determined as described in Section 3.2.2.

#### 3.2.4. Regeneration of Shoots from Roots

During the cultivation of all root clones, it was noted that the roots were able to spontaneously regenerate shoots. This phenomenon prompted an investigation of shoot regenerant cultures. The regeneration of shoots from untransformed and hairy roots was performed on solid DCR or SH media. Five 2–3 cm long root fragments were placed on the media in five 300 mL Erlenmeyer flasks containing 50 mL of solid DCR or SH media. The cultures were cultivated for four weeks under a 16/8 h (light/dark) photoperiod with light provided by cool-white fluorescent lamps (40 µM/m^2^/s) and at a temperature of 25 ± 1 °C. The regenerated shoots were then excised from root tissue and placed on a solid DCR or SH medium, either plain or supplemented with 0.5 mg/L BAP. Each medium was supplemented with 3% sucrose, and their pH was adjusted to 5.7 prior to autoclaving. The experiments were carried out over three subsequent four-week passages. After this time, the number of regenerants per flask was calculated. Four shoots derived from each root line per flask were placed in each flask, and each passage consisted of five flasks.

#### 3.2.5. Root Cultures in Sprinkle Bioreactor

To increase the culture scale, the RgAR line and two hairy root lines, RgTR7 and RgTR17, all after their third passage, were chosen for subculture in a bioreactor. For cultivation, hormone-free DCR and SH media were used. Each medium was supplemented with 3% sucrose, and their pH was adjusted to 5.7 prior to autoclaving. The batch cultures in the bioreactor lasted five weeks. The culture itself was performed in a 5.0 L (working volume 4.0 L) nutrient sprinkle bioreactor consisting of an immobilized glass cylinder (Büchi, Germany). In its interior, a stainless-steel net (the location of the culture) was situated 20 cm above the bottom of the growth bioreactor vessel, and the bottom of the growth chamber was used as a reservoir for the liquid nutrient medium. A Masterflex L/S peristaltic pump (Cole-Parmer, Vernon Hills, IL, USA) provided recirculation and the dosage of the liquid nutrient medium through a polypropylene dispersal nozzle situated 100 mm from the bottom of the growth cylinder. In a single cycle, the medium sprinkled cultivated plant material and was then returned to the medium reservoir. The operating time of the pump for medium delivery was set at 40 s, with 80 s breaks between each delivery; this was later changed to 15 s of medium delivery with 30 s breaks between each delivery. The nutrient was maintained at a constant temperature of 24 ± 1 °C by a Thermamix thermostat (Sartorius BBI Systems GmbH, Melsungen, Germany). The roots were grown in the dark. After five weeks of culture, the plant material was harvested, and the fresh and dry weight (g per bioreactor) was measured.

### 3.3. PCR Analysis

#### 3.3.1. The Confirmation of Transformation

Successful transformation of the roots and root-derived shoots was confirmed at the molecular level by the determination of the T-DNA fragment of the Ri plasmid in the plant tissue by polymerase chain reaction (PCR). The Ri plasmid was isolated from 24 h cultures of the *A. rhizogenes* ATCC 15834 strain using the Plasmid Mini AX Kit (A&A Biotechnology, Gdańsk, Poland) according to the manufacturer’s instructions. The oligonucleotide primers for PCR detection of the homologous sequences to *rolB* (5′-GCTCTTGCAGTGCTAGATTT-3′ and 5′-GAAGGTGCAAGCTACCTCTC-3′) and *rolC* (5′-CTCCTGACATCAAACTCGTC-3′ and 5′-TGCTTCGAGTTATGGGTACA-3′) were designed by Królicka et al. [78]. In addition, whether the investigated organs were free of *A. rhizogenes* was investigated using primers homologous to *virG* (5′-ACTGAATATCAGGCAACGCC-3′ and 5′-GCGTCAAAGAAATAGCCAGC-3′) [83]; although *virG* is present in the Ri plasmid, it is not transferred with T-DNA to the plant cell [84]. The PCR reaction was performed with the Phire^®^ Plant Direct PCR Kit (Thermo Scientific, Waltham, MA, USA) using the dilution protocol specified by the manufacturers. PCR analysis was carried out as follows: one cycle of initial denaturation at 98 °C for 5 min followed by 35 cycles at 98 °C for 5 s, 62 °C for 5 s, and 72 °C for 20 s and a final extension at 72 °C for 1 min. The Thermal Cycler Vario (Applied Biosystems, by Life Technologies, Austin, TX, USA) was used for amplification. The amplification products were resolved by electrophoresis on a 1% agarose gel (Prona, Burgos, Spain) with 1X TBE buffer, stained with SimplySafe (EURx, Gdańsk, Poland), and visualized under UV light (Gel-X Gel Analysis Systems, STI, Poznań, Poland). The size of amplification products was estimated for a molecular weight marker GeneRulerTM 1 kb Plus DNA ladder (Thermo Scientific, Waltham, MA, USA).

#### 3.3.2. Genetic Analysis Using Random Amplified Polymorphic DNA (RAPD) and Start Codon Targeted (SCoT) Markers

The genetic diversity of the in vitro cultivated donor plantlets, and both the non-transformed and transformed shoot regenerants cultivated on the DCR medium with 0.5 mg/L BAP, was confirmed using random amplified polymorphic DNA (RAPD) primers and start codon targeted (SCoT) markers according to Collard and Mackill [85]. The genetic diversity of all investigated root lines was also estimated. The RAPD analysis used a set of 20 arbitrary primers (OPA, Operon Technologies Inc., Alameda, CA, USA) (Table S2), while the SCoT analysis employed 36 arbitrary primers designed by Collard and Mackill [85]. All primers were synthesized by Sigma-Aldrich (Merck KGaA, Darmstadt, Germany). The genomic DNA from the non-transformed and transformed shoot and root tissues was isolated using the CTAB/PVP method developed by Pirttilä et al. [86].

Each PCR reaction (final volume 10 µL) contained: 10X NH_4_ Complete Reaction Buffer, 2 mM dNTP Mix, 0.5 U SuperHotTaq DNA Polymerase (BIORON, Römerberg, Germany), 10 µM OPA/SCoT primers, 10% PVP, 25 mM MgCl_2_, and 25 ng of genomic DNA. All reagents were manufactured by ThermoFisher Scientific (Waltham, MA, USA). The PCR analysis for SCoT primers was performed under the following conditions: initial denaturation at 94 °C for 3 min followed by 35 cycles at 94 °C for 1 min, 51 °C for 1 min, and 72 °C for 2 min and a final extension at 72 °C for 5 min. For the OPA primers (Table S2), PCR analysis was carried out as follows: initial denaturation at 94 °C for 3 min followed by 35 cycles at 94 °C for 1 min, 44 °C for 1 min, and 72 °C for 2 min and a final extension at 72 °C for 7 min. The Thermal Cycler Vario (Applied Biosystems, by Life Technologies, Austin, TX, USA) was used for amplification.

The amplification products, stained with SimplySafe were resolved by electrophoresis on 1.5% agarose gel with 1X TBE buffer and visualized under UV light. The size of amplification products was estimated on the basis of 1 kb and 100 bp DNA ladder molecular weight markers (GeneRulerTM, Thermo Scientific, Waltham, MA, USA). The presence or absence of bands between samples was scored, and bands with the same mobility were counted as identical bands. DNA bands characterized by low visual intensity and those that could not be distinguished as present or not were considered ambiguous markers and were not scored. The resulting bands were scored as present (1) or absent (0) and entered in a computer file as a binary matrix, one for each molecular marker. Then, the matrices were analyzed using FreeTree software [87]. The Dice similarity coefficient was calculated and expressed as a percentage [87]. Hierarchical grouping was performed according to coefficients using the unweighted pair group method of arithmetic means (UPGMA); a UPGMA dendrogram was drawn using STATISTICA 13.1 PL software.

### 3.4. Phytochemical Investigation

All chemicals and reagents, if not stated otherwise, were purchased from Sigma-Aldrich (Merck KGaA, Darmstadt, Germany) or Avantor Performance Materials Poland S.A. (Gliwice, Poland). The standard compounds of lithospermic acid (LA) and lithospermic acid B (LAB) were manufactured by Phyto-Lab and purchased from LCG Standards (Poland); rosmarinic acid (RA) and caffeic acid were purchased from Sigma-Aldrich (Merck KGaA, Darmstadt, Germany).

#### 3.4.1. Sample Preparations

Lyophilized and powdered plant material was used for the phytochemical investigations.

For the HPLC analysis, 100 mg of plant material dry mass was used. The plant material was extracted with 1 mL of 100% methanol for 15 min using an ultrasonic bath (Sonorex Bandelin, Berlin, Germany), then placed on an INFORS gyratory shaker (105 rpm) and incubated overnight at 23 ± 1 °C in the dark. The samples were then centrifuged, and the supernatant was collected. The extraction step was then repeated, and the supernatants were combined and evaporated until dry. The resulting mixtures were stored at −20 °C for analysis.

#### 3.4.2. HPLC–UV–DAD Analysis of Phenolic Acids

The contents of caffeic acid, RA, LA, and LAB were analyzed by HPLC–UV–DAD. Before the HPLC analysis, the samples were re-dissolved in 100% HPLC-grade methanol (1 mL), and 20 μL was included in the HPLC analysis; the setup comprised a DIONEX (Sunnyvale, CA, USA) system with a UVD 340 S diode array detector and an automated sample injector (ASI-100). A NovaPak Phenyl column (150 × 3.9 mm; Waters, Milford, MA, USA) was used for the separation and determination of the phenolic acids in the plant extracts. Water acidified with 0.04 M H_3_PO_4_ (solvent A), acetonitrile (solvent B), and methanol (solvent C) were used as mobile phases. The gradient elution applied was as follows: 0–3 min—85% A, 7% B, 8% C; 3–13 min—70% A, 15% B, 15% C; 13–30 min—60% A, 20% B, 20% C. Eluting substances were visualized by UV absorption at 220 nm.

#### 3.4.3. LCMS-IT-TOF Analysis

The sample analysis was performed according to the method developed by Naliwajski et al. [27]. A Shimadzu Prominence high-performance liquid chromatograph (HPLC) was used, coupled with an LCMS-IT-TOF mass spectrometer (Shimadzu, Kyoto, Japan), equipped with an ion trap (IT), a time-of-flight (TOF) detector, and an electrospray ionization (ESI) source. Mass spectra were recorded in positive ion mode using LCMSsolution software (Shimadzu, Kyoto, Japan). A Kinetex C_18_ column, 2.6 µm, 2.1 × 100 mm (Phenomenex, Torrance, CA, USA), was used with an injection volume of 3 µL, an oven column temperature of 40 °C, a flow rate of 0.2 mL/min, an analysis duration of 75 min, and PDA detection at wavelengths λ = 200–800 nm. The mobile phase consisted of (A) water with the addition of 0.2% CHCOOH and (B) methanol. The following gradient was applied: 0–10 min 5% B, 10–30 min 5→50% B, 30–35 min 50→50% B, 35–55 min 50→95% B, 55–60 min 95% B, 60–62 min 95→5% B, equilibrium time—13 min in 5% B.

In addition, the following conditions for the mass spectrometer were used: polarity positive and negative, mass range *m*/*z* 100–1000 Da in both modes, ion accumulation time: 10 ms in MS1 experiments and 25 ms in MS2 experiments, interface temperature: 220 °C, heat block temperature: 220 °C, nebulizing gas flow: 1.5 L/min, drying gas pressure: 100 kPa, IS: +4.5 kV, collision energy in MS2 experiments: 25–35%. The calibration mixture was used to calibrate the TOF detector of the LCMS-IT-TOF mass spectrometer.

#### 3.4.4. Determination of Total Phenolic Compound Content (TPC)

TFC was estimated according to Pękal and Pyrzynska [88] with slight modifications. TFC was determined based on aluminum complex formation, while TPC was estimated by a Folin–Ciocalteu assay. Briefly, for the TPC assay, 20 µL of plant extract diluted in 80% methanol was added to a 96-well plate, followed by 40 µL of 0.2 N Folin–Ciocalteu reagent and 160 µL of Na_2_CO_3_. The absorbance was measured after 30 min of incubation at 765 nm. The results were expressed as mg of gallic acid equivalent (GAE)/g DW of plant biomass. Each batch sample was measured in four repetitions, and the mean value was taken. The results were calculated and expressed as mg of gallic acid equivalent (GAE) (mg GAE/g DW) using the regression equation determined from the standard curve: y = 0.0053x + 0.1413, r^2^ = 0.9918. For all assays, an Epoch BioTek (Agilent, Santa Clara, CA USA) microplate reader was used.

### 3.5. Antioxidant Activity Analysis

The DPPH assay was performed according to Fukumoto and Mazza [89] with some modifications. Briefly, 200 µL of 2,2-diphenyl-1-picrylhydrazyl-hydrate (DPPH) was added to each 20 µL plant sample diluted in 80% methanol. The mixture was incubated for 60 min at room temperature in the dark, and the absorbance was measured at 517 nm. The radical scavenging activity was expressed as a percentage of DPPH inhibition, calculated according to the formula:

% Inhibition = [(A0 − As)/A0] × 100%

where A0 is the absorbance of the control (DPPH solution + 80% methanol) and As is the absorbance of the sample.

The ferric-reducing antioxidant power (FRAP) assay was carried out according to Benzie and Strain [90], as modified by Bolanos de la Torre et al. [91]. Briefly, 300 µL of FRAP reagent was added to 10 µL plant samples diluted in 80% methanol. The absorbance of the reaction mixture was then detected at 593 nm after 30 min of incubation at room temperature in the dark. Each batch sample was measured in four repetitions, and the mean value was calculated. The results were calculated using the regression equation determined from the standard curve—y = 816.59x, r^2^ = 0.9997—and expressed as mg of Trolox equivalents (TE)/g DW.

### 3.6. Statistical Analysis

All the analyses were performed with appropriate experimental and analytical replicates. The statistical significance between means was assessed using the Kruskal–Wallis one-way analysis of variance performed with STATISTICA 13.1 PL software. The significance between groups was further estimated using the Mann–Whitney U test. A probability of *p* < 0.05 was considered significant. Pair-wise metabolite–antioxidant effects correlations were calculated by Pearson’s correlation coefficient test.

## 4. Conclusions

The biotechnological approaches developed in the current study establish a platform for the sustainable production of bioactive compounds with respect to the protection of plant kingdom biodiversity, which is one of the most important challenges of current times. Our findings indicate that *Rindera graeca*, a rare endemic plant, could be efficiently micropropagated in in vitro cultures. The highest shoot multiplication rates, 7.2 ± 3 and 7.1 ± 5, were obtained on two modifications of a DCR medium supplemented with 0.5 mg/L BAP or kinetin 0.5 mg/L, respectively. Moreover, the shoot and root cultures of this species, both transformed and non-transformed, could serve as sustainable sources of rosmarinic acid and lithospermic acid B. The production capacity depended on the shoot or root line and the culture conditions (type of medium, flask/bioreactor cultivation). Significantly higher levels of RA and LAB were detected in the shoot cultures than in the root ones. The maximum yield of RA was detected in shoots regenerated from RgAR roots during cultivation in the SH medium in the sprinkle bioreactor, i.e., 30.0 ± 3.2 mg/g DW. Among all the investigated root lines, the highest RA accumulation was determined in RgAR roots cultivated in flasks in the DCR medium (14.4 ± 5.9 mg/g DW). While in the bioreactor, the highest yield of RA was detected in RgAR roots during cultivation in the SH medium (5.6 ± 0.7 mg/g DW). The antioxidant potential ranged from 27.9% to 87.4% (DPPH radical scavenging) and from 0.3 to 2.3 µM TE/g DW (FRAP assay), depending on the shoot/root line and the medium used. The antioxidant capacity of the plant extracts did not depend on the content of the investigated phenolic acids but presumably would be affected by the presence of other secondary metabolites, e.g., other phenolic compounds. Genetic analysis carried out using RAPD and SCoT markers demonstrated genetic variability, which reflects the biosynthetic capacity of the cultivated shoot and root lines obtained via biotechnological methods. Our findings demonstrate that the conditions of the in vitro cultures significantly influenced the chemical profile of the investigated phenolic acids in cultivated organs. Furthermore, in vitro *R. graeca* cultures could be considered a source of both rosmarinic acid and lithospermic acid B. Nevertheless, further detailed investigations are needed, e.g., the identification of molecular targets influencing biosynthesis efficiency.

## Figures and Tables

**Table 1:** Summary of the most efficient culture conditions elaborated for micropropagation of various species of the Boraginaceae family.

Plant Species	Culture Conditions for the Highest Shoot Proliferation	Reference
*Arnebia euchroma*	LS medium supplemented with 1 mg/L TDZ: 8.6 shoots per cotyledon explant	[32]
*A. euchroma*	MS medium: 7–15 shoots per explant (shoot bud) cultivated in a sequence of transfer from 20 µM TDZ (10 days) to 5 µM TDZ (20 days)	[33]
*Echium orientale*	MS medium containing 1 mg/L TDZ and 0.5 mg/L IAA, leafy and petiole explants: 8.7 shoots per explant	[34]
	MS medium without any phytohormones: the highest root formation	
*A. benthamii*	MS medium with 3 µM TDZ and 1.5 µM IAA: 20.1/explant (shoot tip)	[35]
*A. hispidissima*	MS medium supplemented with 0.5 mg/L BAP: 17.2 shoots per nodal explant	[36]
	1 mg/L indole-3-butyric acid (IBA) was the most effective for root induction	
	MS medium with addition of 0.5 mg/L kinetin, 0.25 mg/L BAP, 0.1 mg/L IAA, and 100 mg/L casein hydrolysate: cluster of multiple shoots developing from shoot tip and nodal segment; in addition, newly formed shoots not only appeared on the cut edge of the explant but also covered their surface	[37]
*Lithospermum erythrorhizon*	LS medium supplemented with a combination of 0.5 mg/L kinetin and 2 mg/L BAP; the highest shoot multiplication was from shoot segments	[38]
*L. canescens*	LS medium supplemented with a combination of 0.5 mg/L kinetin and 2 mg/L BAP: 6.2 newly developed auxiliary shoots per explant (i.e., shoot tips or shoot fragments with one node)	[39]
*Onosma paniculata*	LS medium supplemented with a combination of 0.5 mg/L kinetin and 2 mg/L BAP: 3 newly developed auxiliary shoots per explant (i.e., shoot tips or shoot fragments with one node)	

**Table 2:** Phenolic acid content (mg/g DW) in shoots and roots of *Rindera graeca* cultivated in vitro.

		**Flask Cultures**							
		**Rosmarinic Acid**				**Lithospermic Acid B**			
Shoot line		Medium							
		DCR	DCR BAP	SH	SH BAP	DCR	DCR BAP	SH	SH BAP
	RgS	na	12.7 ± 4.0	na	na	na	22.5 ± 5.6 ^a^	na	na
	RgAR	14.3 ± 4.0 ^a^	11.9 ± 1.5 *	19.2 ± 3.0 *	17.6 ± 1.6 *	15.3 ± 8.1 *	49.3 ± 15.5 ^a,^*	20.8 ± 9.4	27.9 ± 2.2 ^a^
	RgCR/NOA	12.9 ± 4.6 ^a,^*	14.7 ± 3.4	22.0 ± 4.8 *	19.3 ± 2.3 *	8.6 ± 1.1 *	9.9 ± 0.5 ^a^	12.3 ± 2.6 *	9.2 ± 0.8 ^a,^*
	RgTR7	24.6 ±5.3 ^a,^*	8.6 ± 1.4 *	16.6 ± 2.4 *	14.7 ± 1.0 *	9.7 ± 1.2 *	15.8 ± 11.9 ^a,^*	11.4 ± 2.2 ^a^	11.6 ± 1.4 ^a,^*
	RgTR17	15.3 ± 5.4 ^a,^*	10.0 ± 1.0 *	21.7 ± 1.8 *	16.7 ± 2.6	9.3 ± 3.5	9.4 ± 1.1 ^a^	15.3 ± 1.7 ^a,^*	11.0 ± 2.3 ^a,^*
Root line		Medium							
		DCR		SH		DCR		SH	
	RgAR	14.4 ± 5.9 ^a,*^		1.2 ± 0.4 ^a,^*		0.3 ± 0.1 *		8.5 ± 2.1 ^a,^*	
	RgCR/NOA	10.0 ± 2.5 *		2.5 ± 1.4 ^a,^*		0.2 ± 0.03 *		20.5 ± 5.5 ^a,^*	
	RgTR7	7.4 ± 1.7 ^a^		2.6 ± 1.5 ^a,^*		0.3 ± 0.06 *		23.1 ± 6.6 ^a,^*	
	RgTR17	8.2 ± 1.2 ^a^		2.4 ± 0.9 ^a,^*		0.3 ± 0.06 *		28.9 ± 7.7 ^a^,*	
		**Bioreactor cultures**							
		**Rosmarinic acid**				**Lithospermic Acid B**			
		DCR		SH		DCR		SH	
Shoots regenerated from RgAR roots		na		30.0 ± 3.2 ^a,b^		na		13.1 ± 1.7 ^a^	
RgAR roots		na		5.6 ± 0.7 ^a,b^		na		0	
Shoots regenerated from RgTR17 roots		na		11.6 ± 0.9 ^a,c^		na		3.9 ± 0.2 ^a^	
RgTR17 roots		2.6 ± 0.2 ^a^		2.1 ± 0.1 ^a,c^		0.4 ± 0.01		0	
RgTR7 roots		3.4 ± 0.3 ^a^		na		0		na	

Data represent means ± SD. Differences within the group denoted with the same letter are statistically significant (*p* < 0.05); asterisks denote statistically significant differences between shoot/root lines cultivated in different media; na—not applicable. The concentration of BAP was 0.5 mg/L.

**Table 3:** Antioxidant capacity of *Rindera graeca* shoot and root extracts.

**Shoot line**	**TPC (mg GAE/g DW)**				**FRAP (µM TE/g DW)**				**DPPH (Inhibition %)**			
	Medium											
	DCR	DCR BAP	SH	SH BAP	DCR	DCR BAP	SH	SH BAP	DCR	DCR BAP	SH	SH BAP
RgS	na	24.9 ± 8.8 ^a^	na	na	na	1.7 ± 0.2	na	na	na	76.3 ± 7.5 ^a,b^	na	na
RgAR	53.5 ± 4.8 ^a^	34.7 ± 5.0	195.5 ± 27.2 ^a^	150.5 ± 27.2 ^a^	1.4 ± 0.1	0.3 ± 0.1	1.7 ± 0.3	1.6 ± 0.3 ^a^	57.5 ± 7.7 ^a^	56.0 ± 9.6 ^a,b^	43.6 ± 3.8 ^a,c^	73.0 ± 4.5 ^a^
RgCR/NOA	46.4 ± 8.7 ^a,b^	33.7 ± 4.1	178.8 ± 27.2 ^b^	264.7 ± 32.8 ^a,b^	1.5 ± 0.2	0.3 ± 0.1	1.6 ± 0.4	2.3 ± 0.4 ^a,b^	28.8 ± 9.7 ^a^	72.7 ± 8.8 ^b,c^	58.7 ± 9.0 ^b,c^	73.0 ± 8.8 ^a^
RgTR7	50.3 ± 5.5 ^c^	36.9 ± 5.5 ^a^	174.9 ± 30.2 ^a,b^	187.7 ± 22.8 ^b,c^	1.4 ± 0.1	0.3 ± 0.1	1.6 ± 0.3	1.8 ± 0.2 ^b,c^	27.9 ± 6.7 ^a^	51.1 ± 17.2 ^a,c^	66.5 ± 9.6 ^a,b^	81.2 ± 8.5 ^a^
RgTR17	62.0 ± 5.3 ^a,b,c^	37.6 ± 4.9 ^a^	168.9 ± 16.8	177.9 ± 39.8 ^b,c^	1.5 ± 0.2	0.3 ± 0.1	1.5 ± 0.2	1.8 ± 0.4 ^a,b,c^	35.2 ± 3.6 ^a^	39.9 ± 13.7 ^a,b,c^	58.8 ± 4.5 ^a,b,c^	85.6 ± 4.9 ^a^
**Root line**	**TPC (mg GAE/g DW)**				**FRAP (µM TE/g DW)**				**DPPH (inhibition %)**			
	Medium											
	DCR		SH		DCR		SH		DCR		SH	
RgAR	14.4 ± 2.5 ^a^		45.7 ± 8.1		0.5 ± 0.07 ^a^		0.9 ± 0.1 ^a^		87.4 ± 1.1 ^a^		38.2 ± 8.6	
RgCR/NOA	14.1 ± 0.7 ^b^		44.2 ± 8.1		0.5 ± 0.03 ^a^		0.9 ± 0.1 ^b^		86.8 ± 0.7 ^b^		44.6 ± 9.6	
RgTR7	10.8 ± 2.9		37.1 ± 8.6		0.5 ± 0.1 ^a^		0.8 ± 0.1 ^a,b,c^		86.5 ± 1.2		36.1 ± 9.2	
RgTR17	6.9 ± 0.9 ^a,b^		40.3 ± 9.5		0.3 ± 0.03 ^a^		0.9 ± 0.1 ^c^		83.8 ± 2.4 ^a,b^		32.8 ± 6.9	

DCR BAP—DCR medium supplemented with 0.5 mg/L BAP; SH BAP—SH medium supplemented with 0.5 mg/L BAP. Means ± SD within groups denoted with the same letter are statistically significant (*p* < 0.05); na—not applicable.

## Data Availability

The data that support the findings of this study are available from the corresponding author upon reasonable request.

## Supplementary Materials

The following supporting information can be downloaded at: https://www.mdpi.com/article/10.3390/molecules28124880/s1, Figure S1: Shoots regenerated from root explants of RgCR/NOA and RgTR17 root lines cultivated on various solid media; Table S1: Auxiliary shoot formation of *Rindera graeca* explants derived from seedlings after 4 weeks of culture on various modifications of DCR medium; Table S2: OPA primer sequence used to estimate genetic diversity of cultured in vitro shoot and root lines of *Rindera graeca* in RAPD reaction; Table S3: Dice similarity coefficient [%]; Table S4: Mass spectra of rosmarinic acid and lithospermic acid B detected in examined extracts of shoot and root *Rindera graeca* lines.
