# Supplementary material for: Rindera graeca (A. DC.) Boiss. & Heldr. (Boraginaceae) In Vitro Cultures Targeting Lithospermic Acid B and Rosmarinic Acid Production
Source: Molecules. 2023 Jun 20;28(12):4880. doi: 10.3390/molecules28124880 (PMC10303423; doi:10.3390/molecules28124880)
Supplement: Supplementary file 1 [file molecules-28-04880-s001.zip › Figure S1.pdf]

Rindera graeca (A. DC.) Boiss. & Heldr. (Boraginaceae) in vitro cultures targeting lithospermic acid B and rosmarinic acid production

Katarzyna Sykłowska-Baranek [1]*, Małgorzata Gaweł [1], Łukasz Kuźma [2], Beata Wileńska [3,4], Mateusz Kawka [1], Małgorzata Jeziorek [1], Konstantia Graikou [5], Ioanna Chinou [5], Ewa Szyszko [1], Piotr Stępień [1], Patryk Zakrzewski [1] and Agnieszka Pietrosiuk [1]

[1] Department of Pharmaceutical Biology, Faculty of Pharmacy, Medical University of Warsaw, 1 Banacha St., 02-097 Warsaw, Poland; mgawel1@wum.edu.pl (M.G.); mateusz.kawka@wum.edu.pl (M.K.); mjeziorek@wum.edu.pl (M.J.); pryzmik16@op.pl (E.S.); piotrste1@wp.pl (P.S.); patrol20013@gmail.com (P.Z.); agnieszka.pietrosiuk@wum.edu.pl (A.P.)

[2] Department of Biology and Pharmaceutical Botany, Faculty of Pharmacy, Medical University of Łódź, 1 Muszyńskiego, 90-151 Łódź, Poland; lukasz.kuzma@umed.lodz.pl

[3] Faculty of Chemistry, University of Warsaw, 1 Pasteura St., 02-093 Warsaw, Poland;

[4] Biological and Chemical Research Centre, 101 Żwirki i Wigury St., 02-097 Warsaw, Poland; bwilenska@chem.uw.edu.pl

[5] Laboratory of Pharmacognosy and Chemistry of Natural Products, Faculty of Pharmacy, National and Kapodistrian University of Athens, Panepistimiopolis, 15771 Athens, Greece; kgraikou@pharm.uoa.gr (K.G.); ichinou@pharm.uoa.gr (I.C.)

* Correspondence: katarzyna.syklowska-baranek@wum.edu.pl

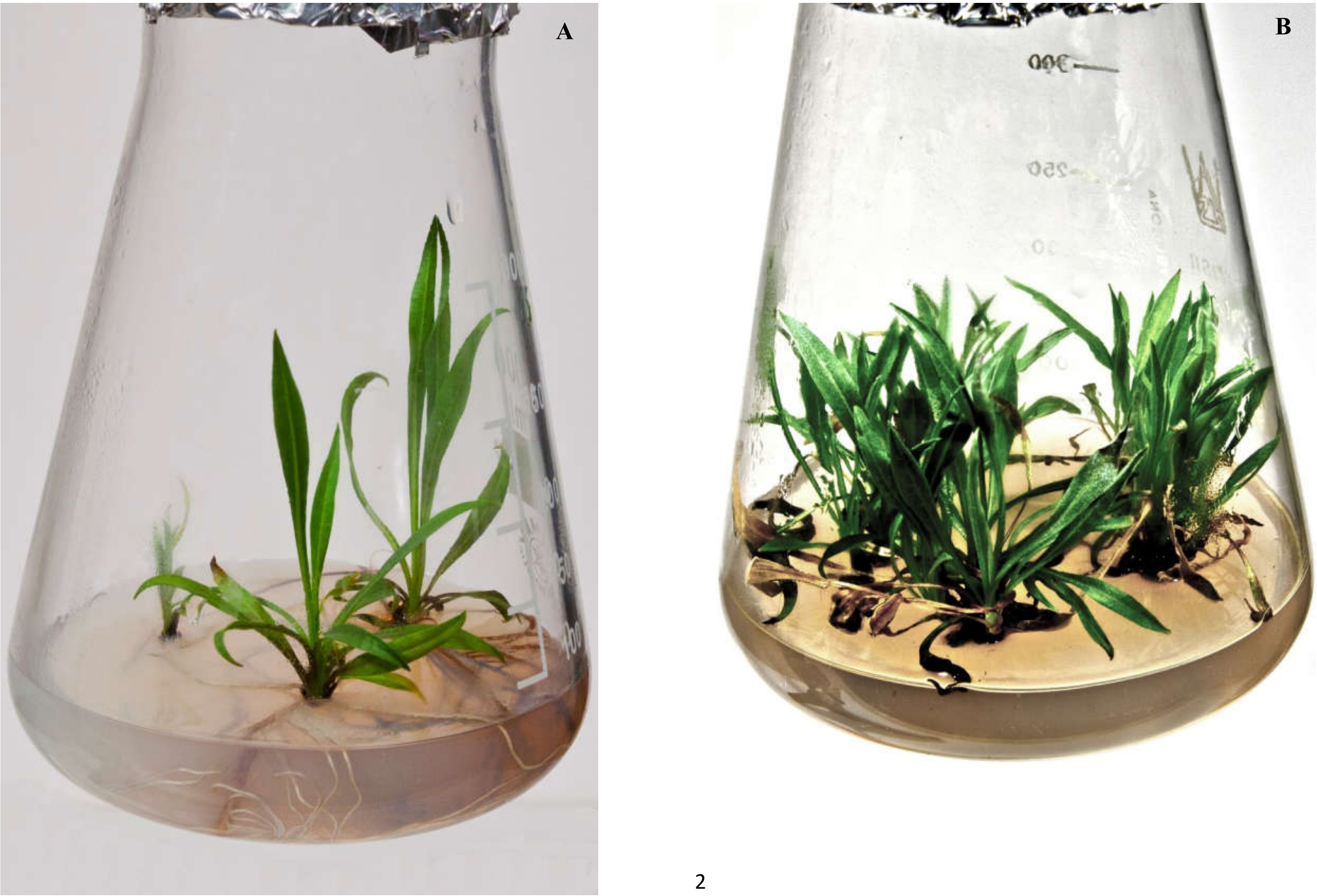
A
B

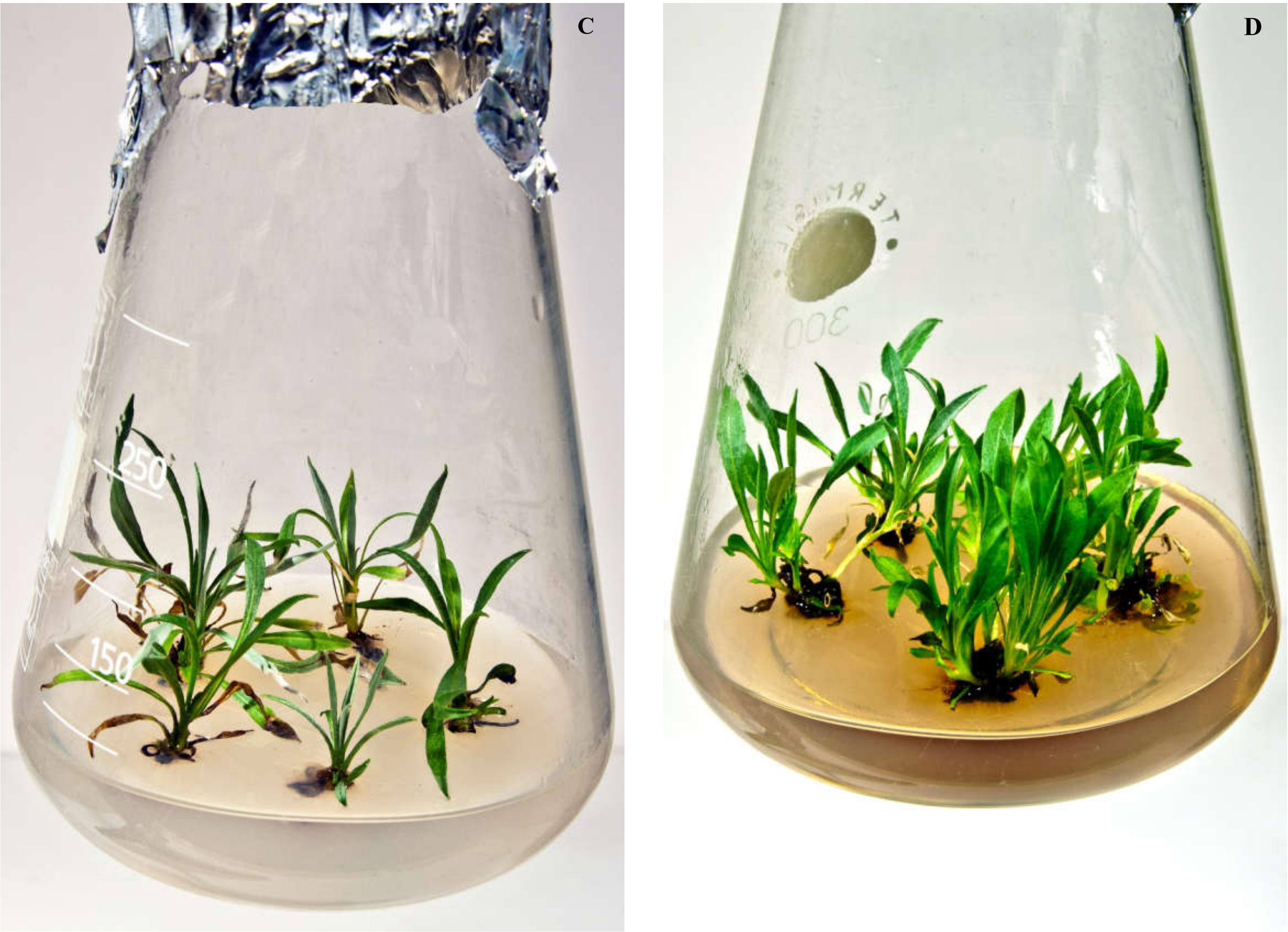
C
250
150
D
300

Figure S1. Shoots regenerated from root explants of RgCR/NOA root line cultivated on solid hormone-free DCR medium (A) or (B) on DCR medium supplemented with 0.5 mg/L BAP; (C) shoots regenerated form explants of RgTR17 hairy root line and cultivated, after separation from roots, for four weeks on hormone-free SH medium or (D) on SH medium supplemented with 0.5 mg/L BAP. The cultures were performed at 16h/8h (light/dark) photoperiod with light provided by cool-white fluorescent lamps (40 $\mu M/m^2/s^1$).
