# Supplementary material for: Rindera graeca (A. DC.) Boiss. & Heldr. (Boraginaceae) In Vitro Cultures Targeting Lithospermic Acid B and Rosmarinic Acid Production
Source: Molecules. 2023 Jun 20;28(12):4880. doi: 10.3390/molecules28124880 (PMC10303423; doi:10.3390/molecules28124880)
Supplement: Supplementary file 1 [file molecules-28-04880-s001.zip › Table S1.pdf]

Rindera graeca (A. DC.) Boiss. & Heldr. (Boraginaceae) in vitro cultures targeting lithospermic acid B and rosmarinic acid production

Katarzyna Sykłowska-Baranek [1*], Małgorzata Gaweł [1], Łukasz Kuźma [2], Beata Wileńska [3,4], Mateusz Kawka [1], Małgorzata Jeziorek [1], Konstantia Graikou [5], Ioanna Chinou [5], Ewa Szyszko [1], Piotr Stępień [1], Patryk Zakrzewski [1] and Agnieszka Pietrosiuk [1]

1 Department of Pharmaceutical Biology, Faculty of Pharmacy, Medical University of Warsaw, 1 Banacha St., 02-097 Warsaw, Poland; mgawel1@wum.edu.pl (M.G.); mateusz.kawka@wum.edu.pl (M.K.); mjeziorek@wum.edu.pl (M.J.); pryzmik16@op.pl (E.S.); piotrste1@wp.pl (P.S.); patrol20013@gmail.com (P.Z.); agnieszka.pietrosiuk@wum.edu.pl (A.P.)

2 Department of Biology and Pharmaceutical Botany, Faculty of Pharmacy, Medical University of Łódź, 1 Muszyńskiego, 90-151 Łódź, Poland; lukasz.kuzma@umed.lodz.pl

3 Faculty of Chemistry, University of Warsaw, 1 Pasteura St., 02-093 Warsaw, Poland;

4 Biological and Chemical Research Centre, 101 Żwirki i Wigury St., 02-097 Warsaw, Poland; bwilenska@chem.uw.edu.pl

5 Laboratory of Pharmacognosy and Chemistry of Natural Products, Faculty of Pharmacy, National and Kapodistrian University of Athens, Panepistimiopolis, 15771 Athens, Greece; kgraikou@pharm.uoa.gr (K.G.); ichinou@pharm.uoa.gr (I.C.)

* Correspondence: katarzyna.syklowska-baranek@wum.edu.pl

Table S1. Auxiliary shoot formation of *Rindera graeca* explants derived from seedlings after 4 weeks of culture on various modifications of DCR medium.

Medium	Growth regulator (mg/L)	Mean shoot number/explant	Shoot description
DCR	BAP 0.5	7.2 ± 3	dark green leaves, luxuriant rosettes
DCR/M	BAP 0.5	5.5 ± 3	light green leaves
DCR/S	BAP 0.5	5.6 ± 4	light green leaves
DCR/SV	BAP 0.5	4.5 ± 3	light green leaves
DCR	BAP 1	4.8 ± 4	yellow-green leaves
DCR/M	BAP 1	3.7 ± 3	yellow-green leaves
DCR/S	BAP 1	6.6 ± 3	yellow green leaves
DCR/SV	BAP 1	5.9 ± 3	yellow green leaves
DCR	KIN 0.5	3.0 ± 2	light green leaves
DCR/M	KIN 0.5	1.8 ± 2	yellow green leaves
DCR/S	KIN 0.5	4.6 ± 3	yellow green leaves
DCR/SV	KIN 0.5	7.1 ± 5	light green leaves
DCR	KIN 1	4.6 ± 3	yellow-green leaves
DCR/M	KIN 1	2.0 ± 3	yellow-green leaves
DCR/S	KIN 1	2.5 ± 2	yellow-green leaves
DCR/SV	KIN 1	3.9 ± 3	yellow-green leaves

Data represents means ± SD.

DCR – full strength DCR medium; DCR/M – DCR medium with reduced to half content of macroelements; DCR/S – DCR medium with reduced to half content of macro- and microelements; DCR/SV - DCR medium with reduced to half content of macro-, microelements and vitamins.
