# Supplementary material for: Rindera graeca (A. DC.) Boiss. & Heldr. (Boraginaceae) In Vitro Cultures Targeting Lithospermic Acid B and Rosmarinic Acid Production
Source: Molecules. 2023 Jun 20;28(12):4880. doi: 10.3390/molecules28124880 (PMC10303423; doi:10.3390/molecules28124880)
Supplement: Supplementary file 1 [file molecules-28-04880-s001.zip › Table S2.pdf]

Rindera graeca (A. DC.) Boiss. & Heldr. (Boraginaceae) in vitro cultures targeting lithospermic acid B and rosmarinic acid production

Katarzyna Sykłowska-Baranek [1*], Małgorzata Gaweł [1], Łukasz Kuźma [2], Beata Wileńska [3,4], Mateusz Kawka [1], Małgorzata Jeziorek [1], Konstantia Graikou [5], Ioanna Chinou [5], Ewa Szyszko [1], Piotr Stępień [1], Patryk Zakrzewski [1] and Agnieszka Pietrosiuk [1]

[11] Department of Pharmaceutical Biology, Faculty of Pharmacy, Medical University of Warsaw, 1 Banacha St., 02-097 Warsaw, Poland; mgawel1@wum.edu.pl (M.G.); mateusz.kawka@wum.edu.pl (M.K.); mjeziorek@wum.edu.pl (M.J.); pryzmik16@op.pl (E.S.); piotrste1@wp.pl (P.S.); patrol20013@gmail.com (P.Z.); agnieszka.pietrosiuk@wum.edu.pl (A.P.)

[2] Department of Biology and Pharmaceutical Botany, Faculty of Pharmacy, Medical University of Łódź, 1 Muszyńskiego, 90-151 Łódź, Poland; lukasz.kuzma@umed.lodz.pl

[3] Faculty of Chemistry, University of Warsaw, 1 Pasteura St., 02-093 Warsaw, Poland;

[4] Biological and Chemical Research Centre, 101 Żwirki i Wigury St., 02-097 Warsaw, Poland; bwilenska@chem.uw.edu.pl

[5] Laboratory of Pharmacognosy and Chemistry of Natural Products, Faculty of Pharmacy, National and Kapodistrian University of Athens, Panepistimiopolis, 15771 Athens, Greece; kgraikou@pharm.uoa.gr (K.G.); ichinou@pharm.uoa.gr (I.C.)

* Correspondence: katarzyna.syklowska-baranek@wum.edu.pl

Table S2. OPA primers sequence used to estimate genetic diversity of cultured *in vitro* shoot and root lines of *Rindera graeca* in RAPD reaction.

Primer name	Primer 5′ 3′sequence
OPA01	CAGGCCCTTC
OPA02	TGCCGAGCTG
OPA03	AGTCAGCCAC
OPA04	AATCGGGCTG
OPA05	AGGGGTCTTG
OPA06	GGTCCCTGAC
OPA07	GAAACGGGTG
OPA08	GTGACGTAGG
OPA09	GGGTAACGCC
OPA10	GTGATCGCAG
OPA11	GTGATCGCAG
OPA12	TCGGCGATAG
OPA13	CAGCACCCAC
OPA14	TCTGTGCTGG
OPA15	TTCCGAACCC
OPA16	AGCCAGCGAA
OPA17	GACCGCTTGT
OPA18	AGGTGACCGT
OPA19	CAAACGTCGG
OPA20	GTTGCGATCC
