# Supplementary material for: Rindera graeca (A. DC.) Boiss. & Heldr. (Boraginaceae) In Vitro Cultures Targeting Lithospermic Acid B and Rosmarinic Acid Production
Source: Molecules. 2023 Jun 20;28(12):4880. doi: 10.3390/molecules28124880 (PMC10303423; doi:10.3390/molecules28124880)
Supplement: Supplementary file 1 [file molecules-28-04880-s001.zip › Table S3.pdf]

Rindera graeca (A. DC.) Boiss. & Heldr. (Boraginaceae) in vitro cultures targeting lithospermic acid B and rosmarinic acid production

Katarzyna Sykłowska-Baranek [1*], Małgorzata Gaweł [1], Łukasz Kuźma [2], Beata Wileńska [3,4], Mateusz Kawka [1], Małgorzata Jeziorek [1], Konstantia Graikou [5], Ioanna Chinou [5], Ewa Szyszko [1], Piotr Stępień [1], Patryk Zakrzewski [1] and Agnieszka Pietrosiuk [1]

[1] Department of Pharmaceutical Biology, Faculty of Pharmacy, Medical University of Warsaw, 1 Banacha St., 02-097 Warsaw, Poland; mgawel1@wum.edu.pl (M.G.); mateusz.kawka@wum.edu.pl (M.K.); mjeziorek@wum.edu.pl (M.J.); pryzmik16@op.pl (E.S.); piotrste1@wp.pl (P.S.); patrol20013@gmail.com (P.Z.); agnieszka.pietrosiuk@wum.edu.pl (A.P.)

[2] Department of Biology and Pharmaceutical Botany, Faculty of Pharmacy, Medical University of Łódź, 1 Muszyńskiego, 90-151 Łódź, Poland; lukasz.kuzma@umed.lodz.pl

[3] Faculty of Chemistry, University of Warsaw, 1 Pasteura St., 02-093 Warsaw, Poland;

[4] Biological and Chemical Research Centre, 101 Żwirki i Wigury St., 02-097 Warsaw, Poland; bwilenska@chem.uw.edu.pl

[5] Laboratory of Pharmacognosy and Chemistry of Natural Products, Faculty of Pharmacy, National and Kapodistrian University of Athens, Panepistimiopolis, 15771 Athens, Greece; kgraikou@pharm.uoa.gr (K.G.); ichinou@pharm.uoa.gr (I.C.)

* Correspondence: katarzyna.syklowska-baranek@wum.edu.pl

Table S3. Dice similaryty coafficient [%].

Shoot/root line	RgAR-shoots	RgAR-roots	RgCR/NOA-shoots	RgCR/NOA-roots	RgTR7 shoots	RgTR7 roots	RgTR17 shoots	RgTR17 roots	RgS shoots
**RgAR-shoots**		68.153	81.955	79.012	89.744	68.831	86.066	62.759	81.481
**RgAR-roots**	68.153		82.123	75.2244	71.779	96.500	75.000		
**RgCR/NOA-shoots**	81.955	82.123		86.411	82.734	81.250	86.111		
**RgCR/NOA-roots**	79.012	75.224	86.411		81.569	74.164	80.755		
**RgTR7 shoots**	89.744	71.779	82.734	81.569		73.125	86.719		
**RgTR7 roots**	68.831	96.500	81.250	74.164	73.125		73.939		
**RgTR17 shoots**	86.066	75.000	86.111	80.755	86.719	73.939			
**RgTR17 roots**	63.514	91.753	76.471	73.186	68.831	91.099	71.069		
**RgS shoots**	81.418	65.584	76.923	74.262	83.333	66.225	82.353		
