# Supplementary material for: Rindera graeca (A. DC.) Boiss. & Heldr. (Boraginaceae) In Vitro Cultures Targeting Lithospermic Acid B and Rosmarinic Acid Production
Source: Molecules. 2023 Jun 20;28(12):4880. doi: 10.3390/molecules28124880 (PMC10303423; doi:10.3390/molecules28124880)

Rindera graeca (A. DC.) Boiss. & Heldr. (Boraginaceae) in vitro cultures targeting lithospermic acid B and rosmarinic acid production

Katarzyna Sykłowska-Baranek [1*], Małgorzata Gaweł [1], Łukasz Kuźma [2], Beata Wileńska [3,4], Mateusz Kawka [1], Małgorzata Jeziorek [1], Konstantia Graikou [5], Ioanna Chinou [5], Ewa Szyszko [1], Piotr Stępień [1], Patryk Zakrzewski [1] and Agnieszka Pietrosiuk [1]

1 [1]Department of Pharmaceutical Biology, Faculty of Pharmacy, Medical University of Warsaw, 1 Banacha St., 02-097 Warsaw, Poland; mgawel1@wum.edu.pl (M.G.); mateusz.kawka@wum.edu.pl (M.K.); mjeziorek@wum.edu.pl (M.J.); pryzmik16@op.pl (E.S.); piotrste1@wp.pl (P.S.); patrol20013@gmail.com (P.Z.); agnieszka.pietrosiuk@wum.edu.pl (A.P.)

2 Department of Biology and Pharmaceutical Botany, Faculty of Pharmacy, Medical University of Łódź, 1 Muszyńskiego, 90-151 Łódź, Poland; lukasz.kuzma@umed.lodz.pl

3 Faculty of Chemistry, University of Warsaw, 1 Pasteura St., 02-093 Warsaw, Poland;

4 Biological and Chemical Research Centre, 101 Żwirki i Wigury St., 02-097 Warsaw, Poland; bwilenska@chem.uw.edu.pl

5 Laboratory of Pharmacognosy and Chemistry of Natural Products, Faculty of Pharmacy, National and Kapodistrian University of Athens, Panepistimiopolis, 15771 Athens, Greece; kgraikou@pharm.uoa.gr (K.G.); ichinou@pharm.uoa.gr (I.C.)

* Correspondence: katarzyna.syklowska-baranek@wum.edu.pl

Table S4. Mass spectra of rosmarinic and lithospermic B acids detected in examined extracts of shoot and root *Rindera graeca* lines.

		**Mass spectra**
**Rosmarinic acid (RA) RT = 26.981**	MS1	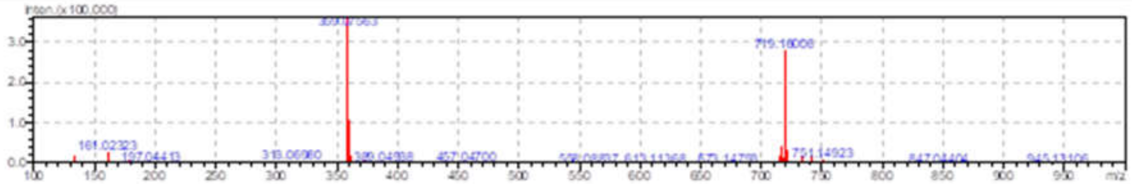
	MS2	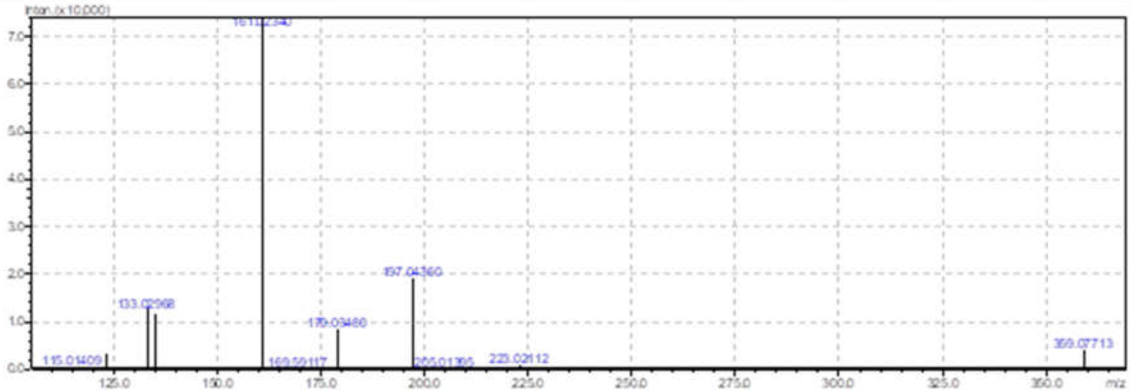
**Lithospermic B acid (LAB) RT = 27.969**	MS1	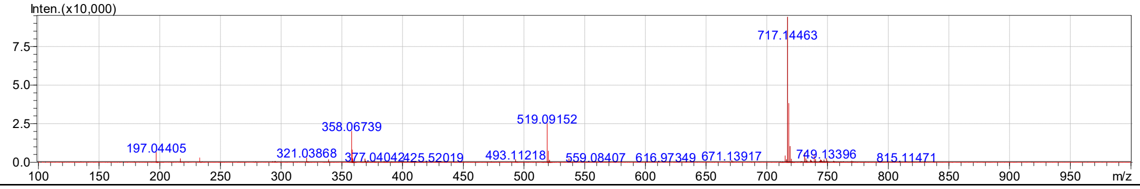

MS2

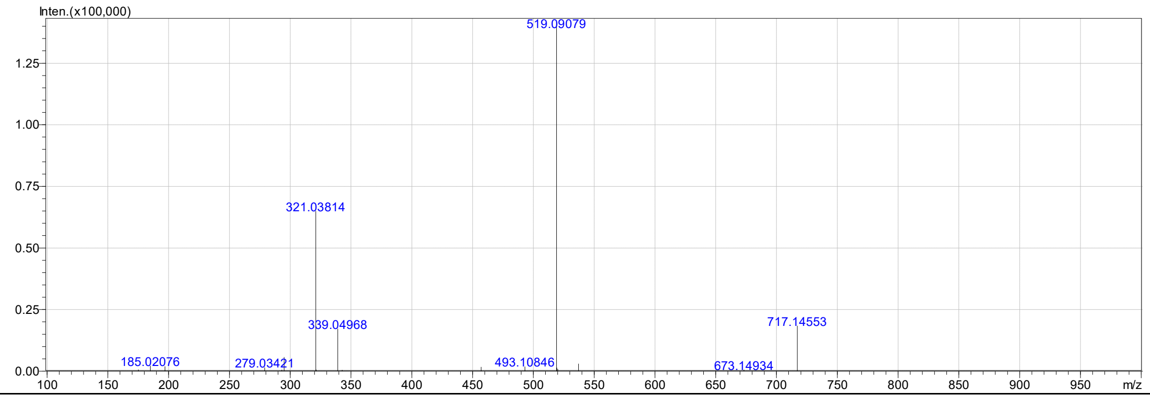

RgS shoots

RA MS1

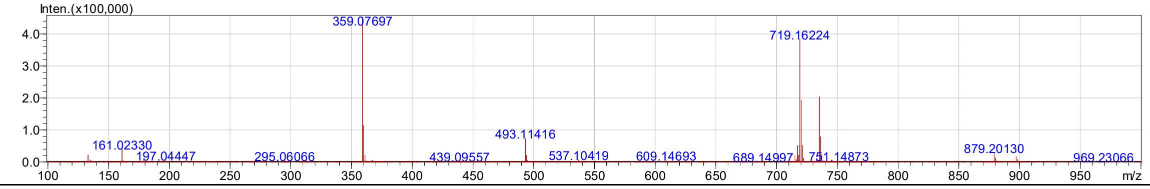

RA MS2

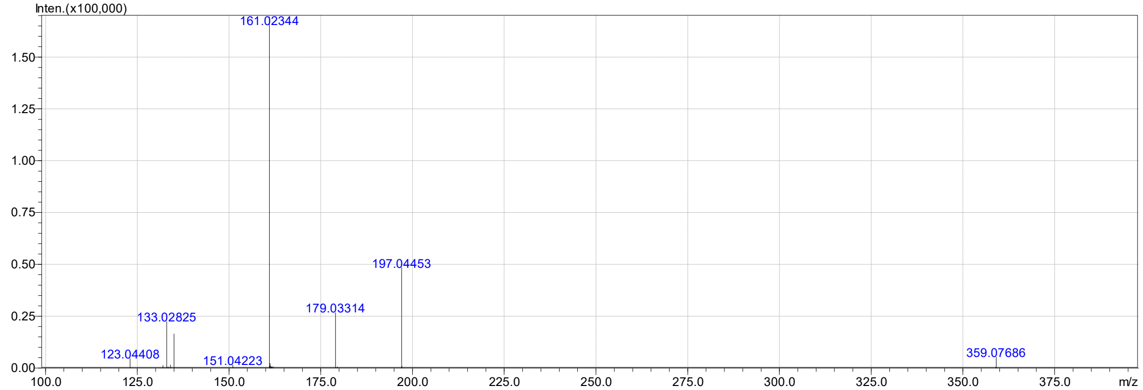

LAB MS1

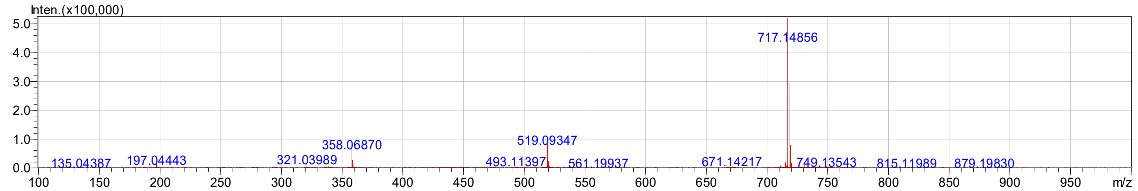

LAB MS2

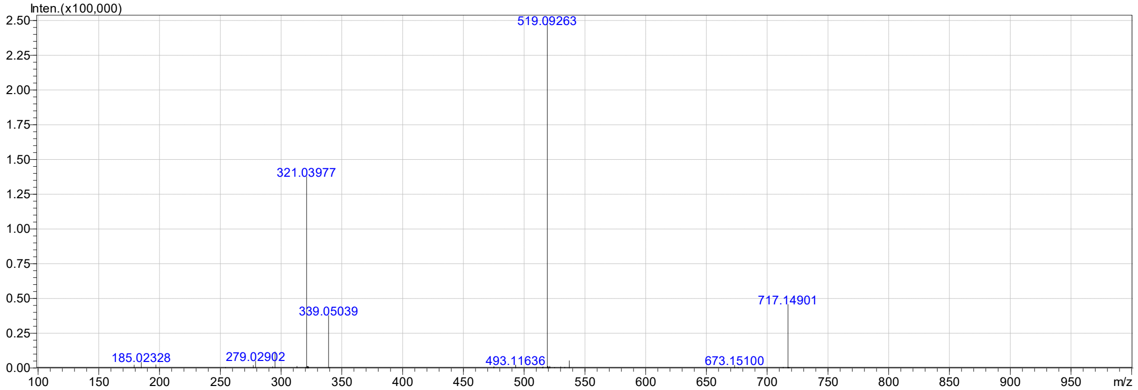

RgAR shoots RA MS1

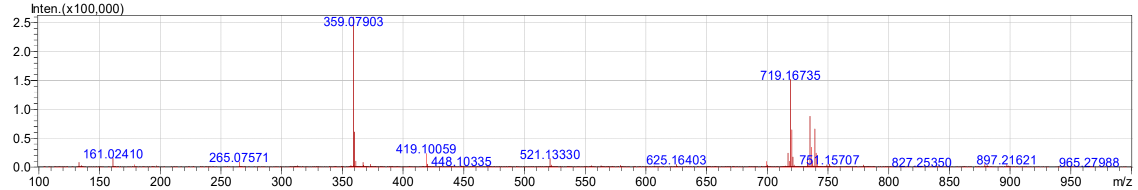

RA MS2

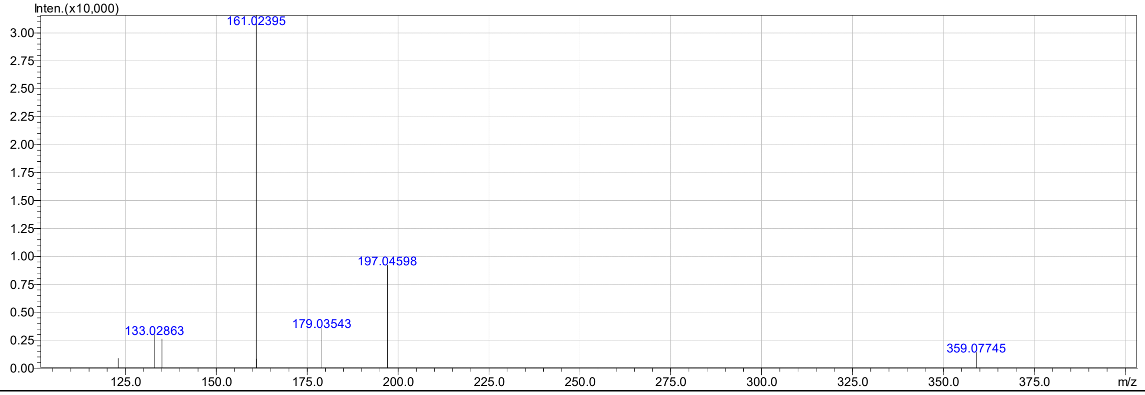

LAB MS1

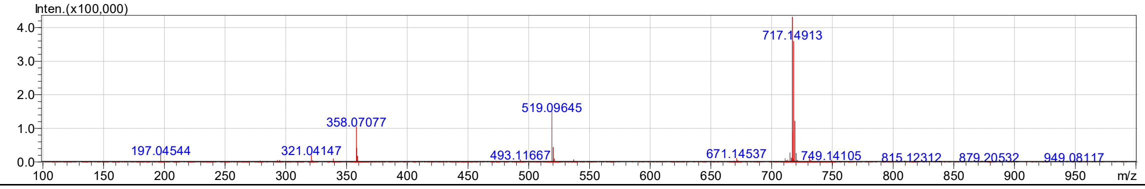

LAB MS2

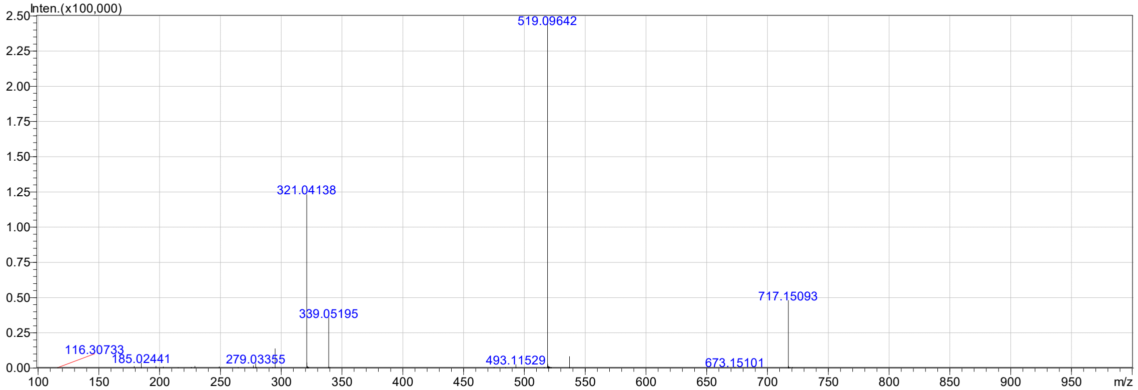

RgCR/NOA shoots

RA MS1

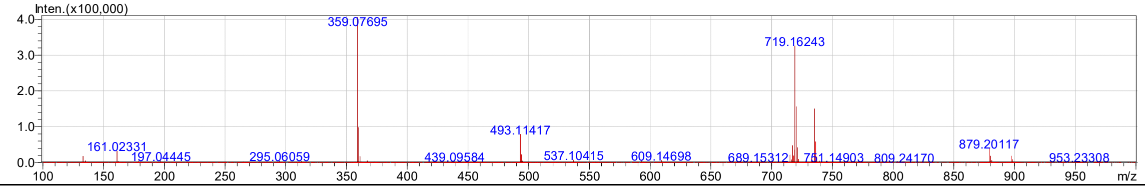

RA MS2

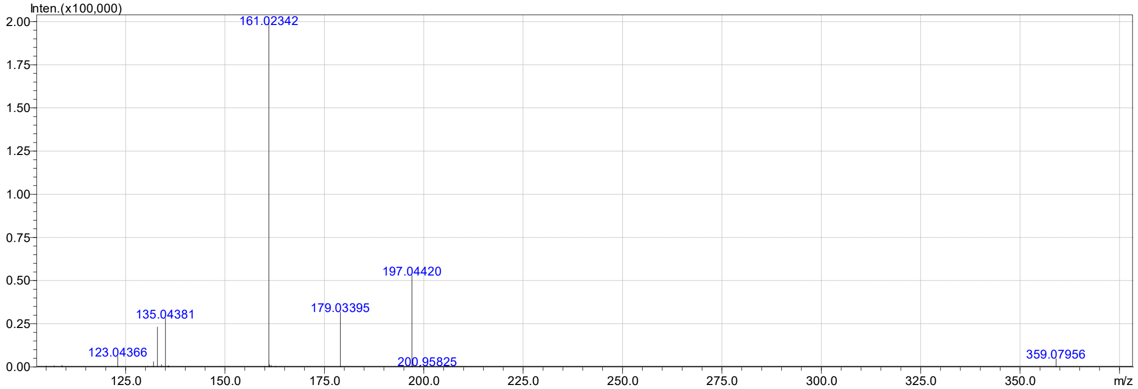

LAB MS1

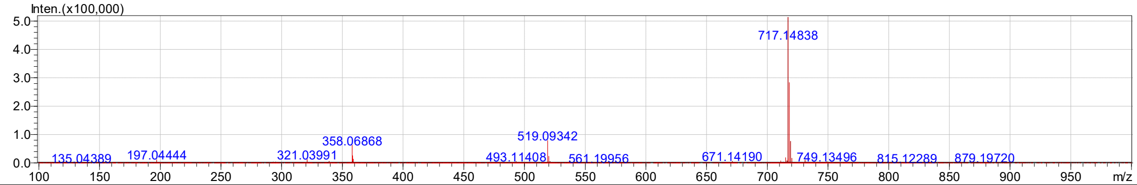

	LAB MS2	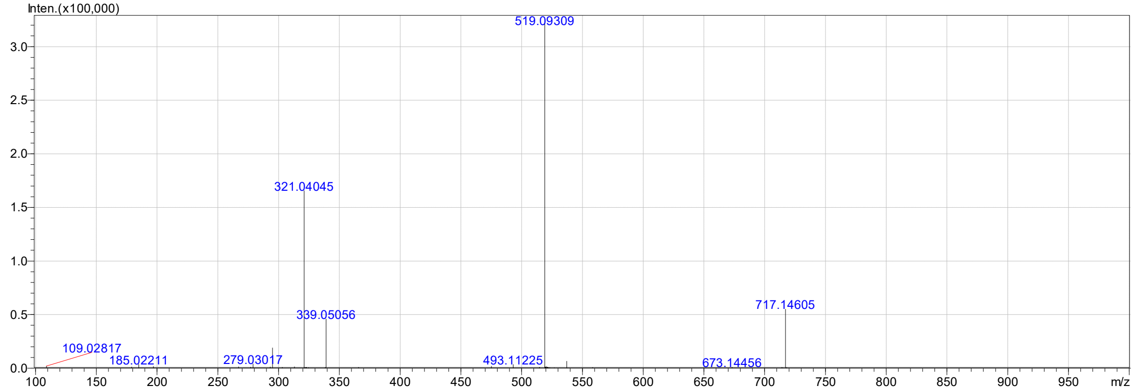
	RA MS1	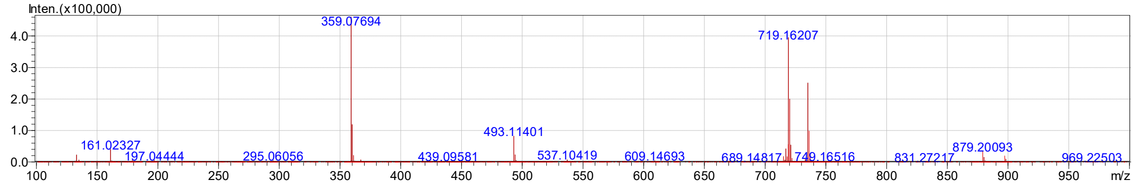
**RgTR7 shoots**	RA MS2	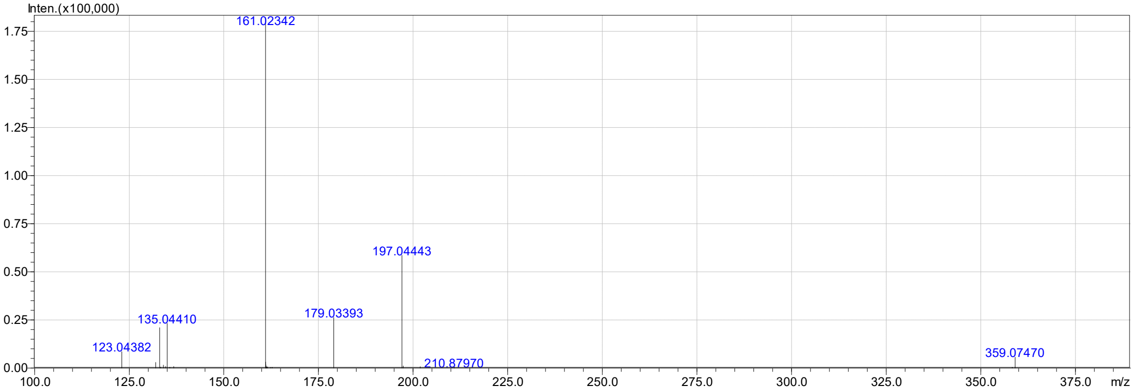

LAB MS1

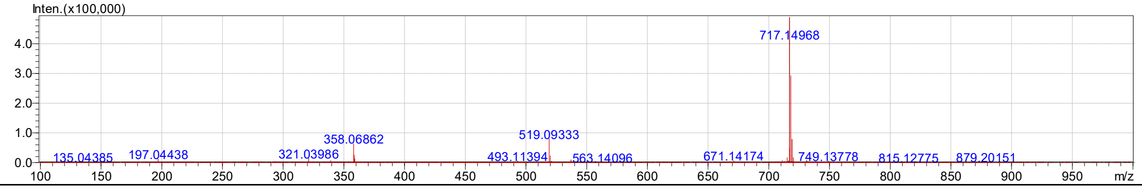

LAB MS2

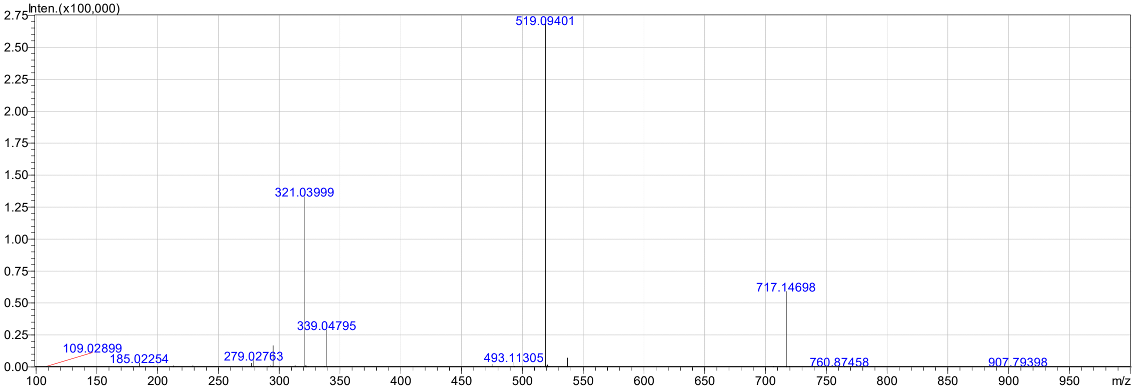

RgTR17 shoots

RA MS1

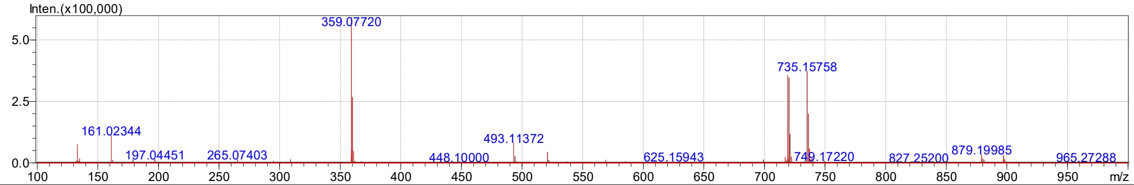

RA MS2

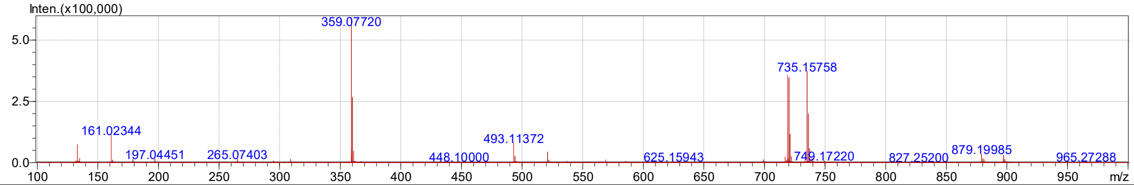

	LAB MS1	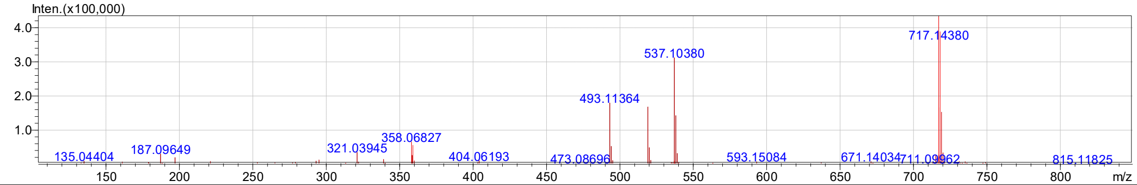
	LAB MS2	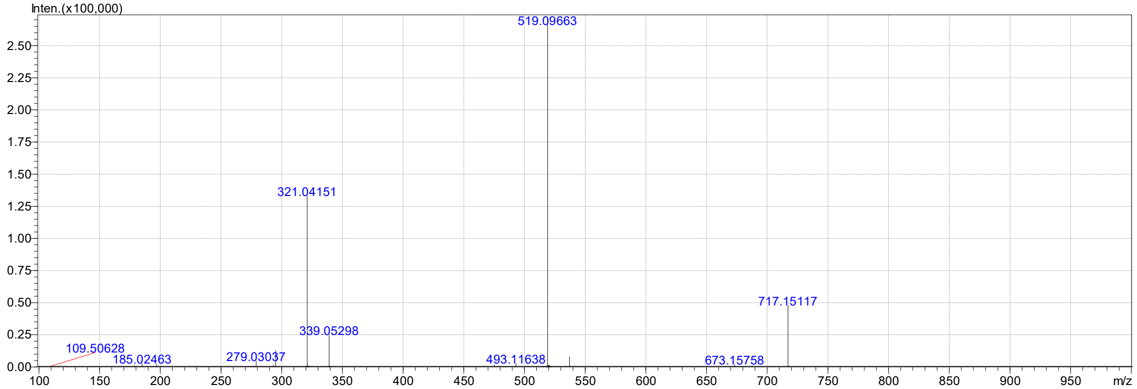
**RgAR roots**	RA MS1	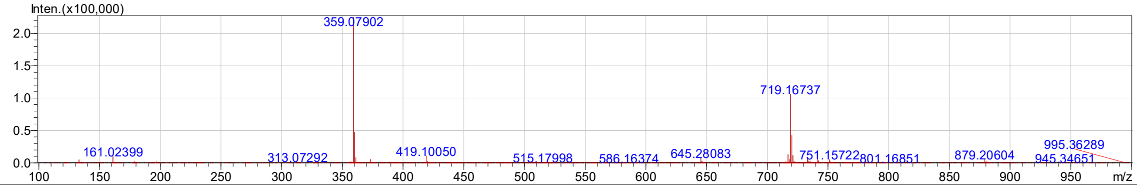

RA MS2

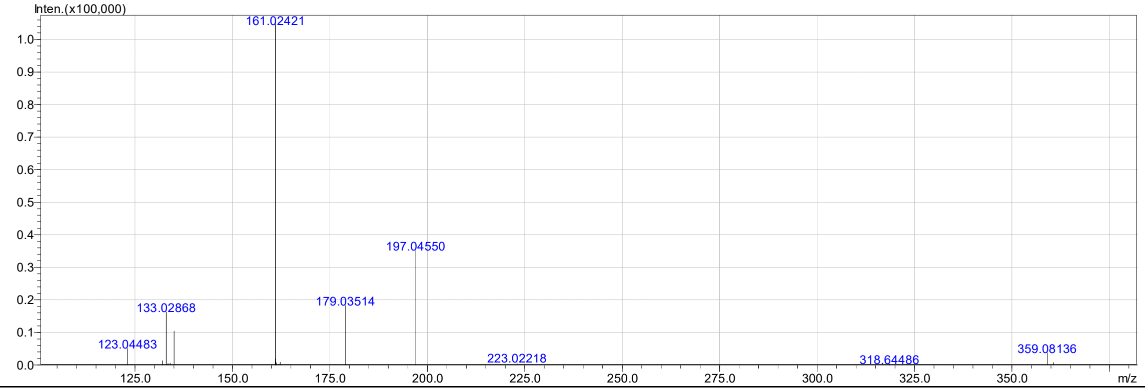

LAB MS1

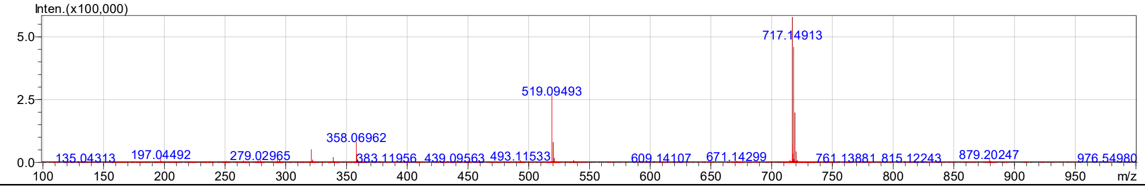

LAB MS2

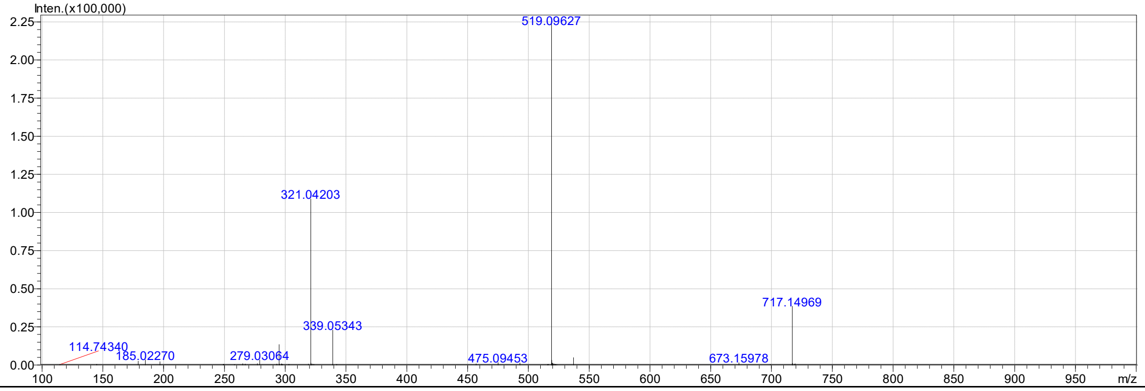

RgCR/NOA roots

RA MS1

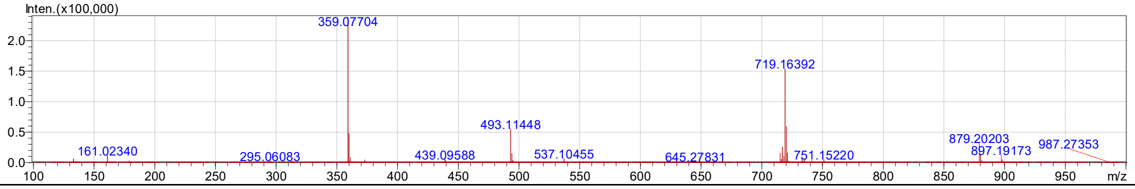

RA MS2

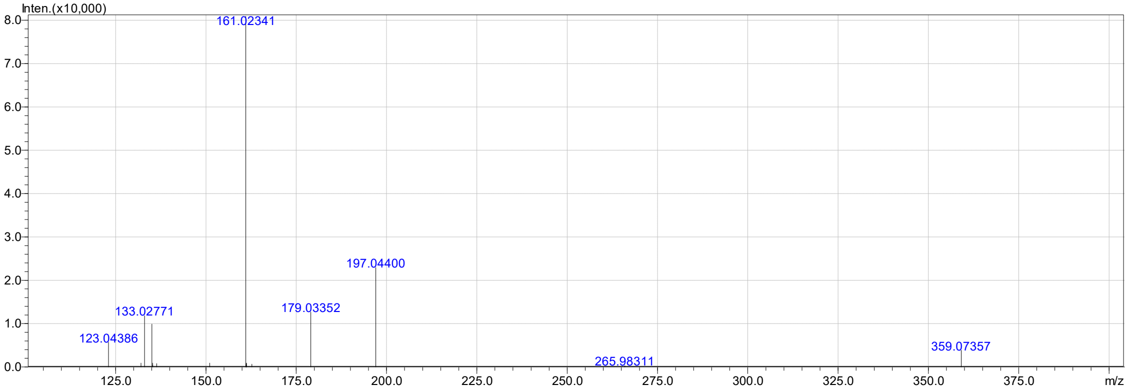

LAB MS1

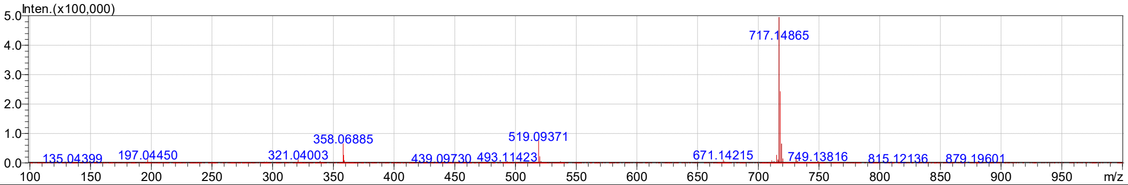

	LAB MS2	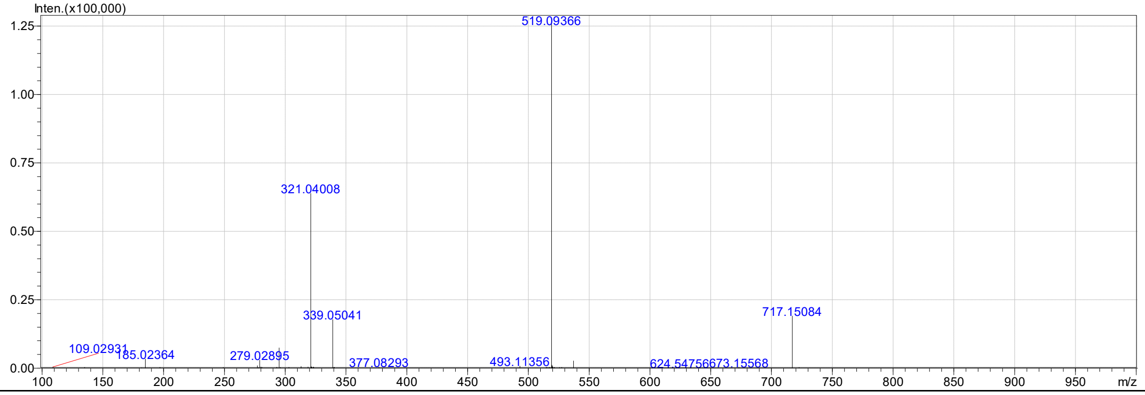
RgTR7 roots	RA MS1	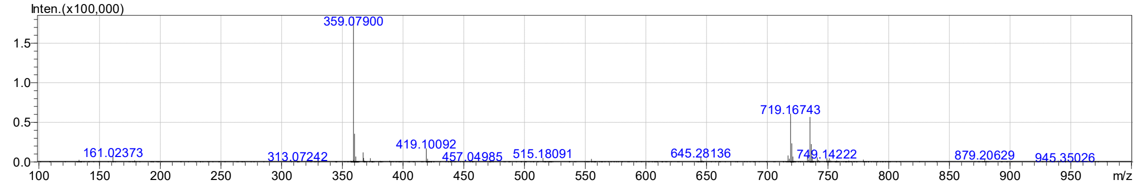
	RA MS2	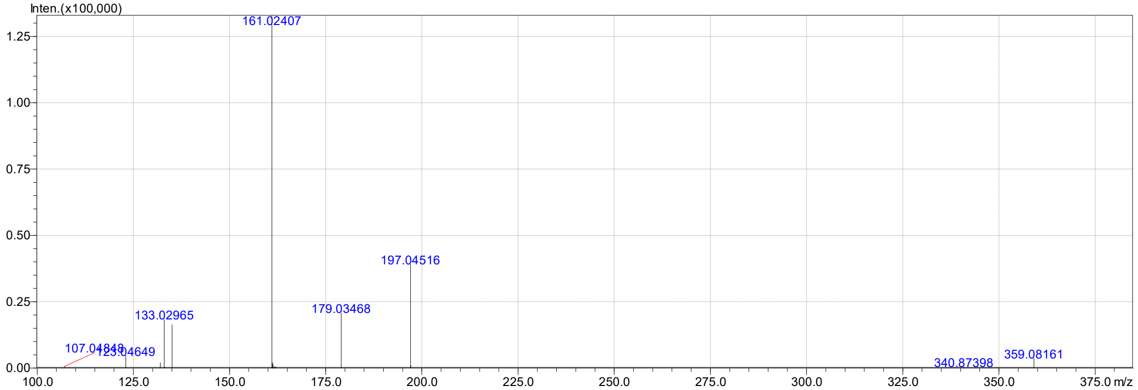

LAB MS1

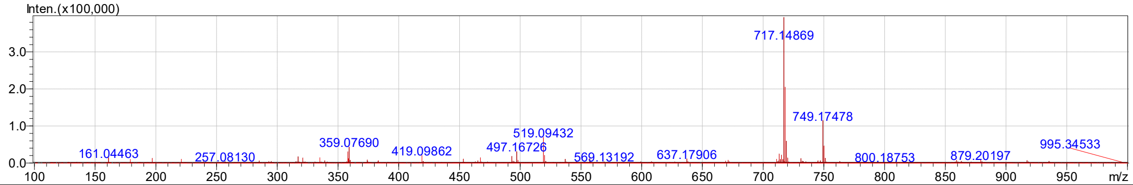

LAB MS2

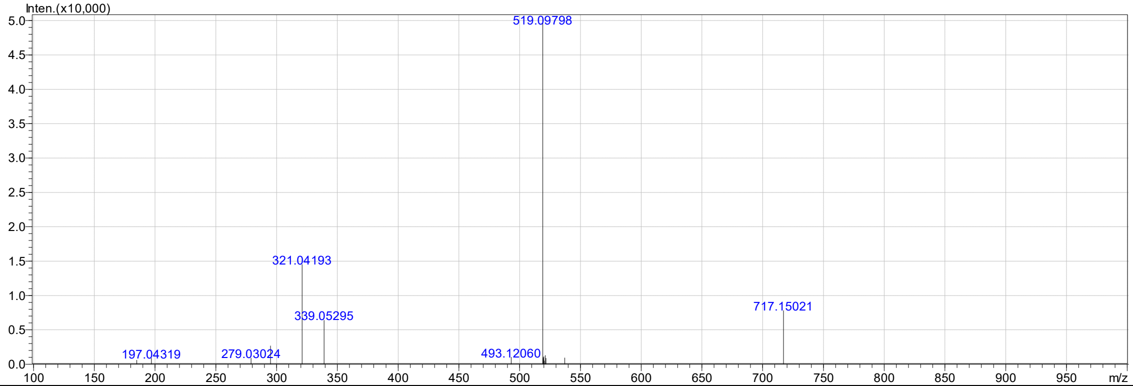

RgTR17 roots

RA MS1

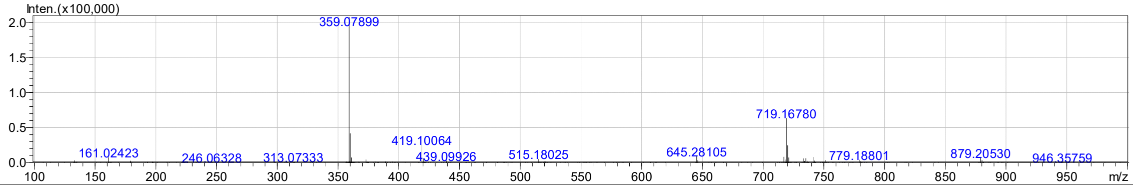

RA MS2

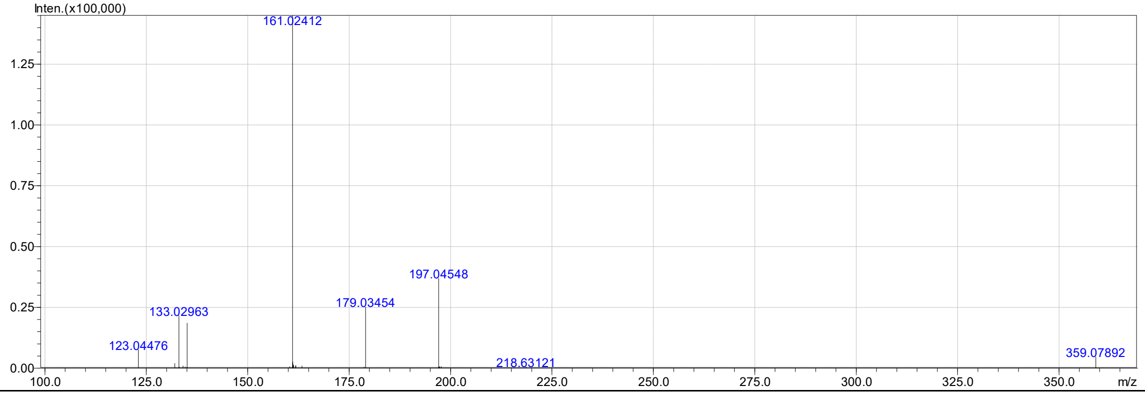

LAB MS1

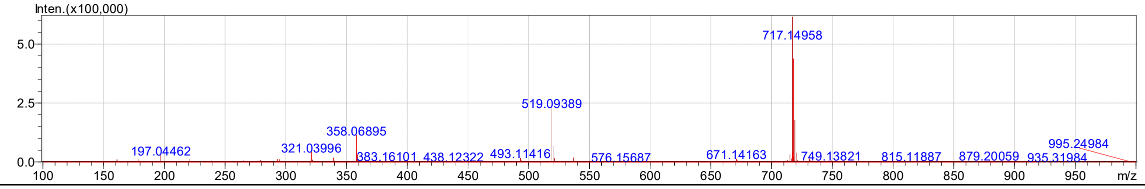

LAB MS2

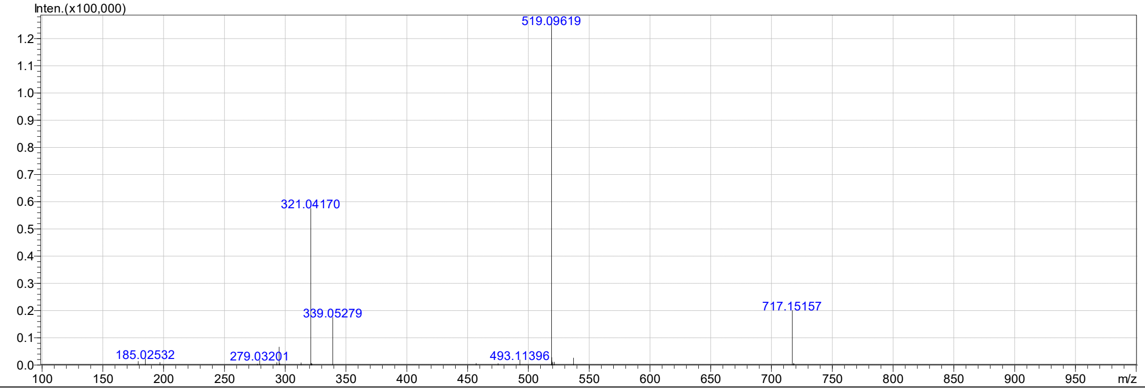

Supplement: Supplementary file 1 [file molecules-28-04880-s001.zip › Table S4.pdf]
